# Where Children Play: Young Child Exposure to Environmental Hazards during Play in Public Areas in a Transitioning Internally Displaced Persons Community in Haiti

**DOI:** 10.3390/ijerph15081646

**Published:** 2018-08-03

**Authors:** Danielle N. Medgyesi, John M. Brogan, Daniel K. Sewell, Jean Philippe Creve-Coeur, Laura H. Kwong, Kelly K. Baker

**Affiliations:** 1 Department of Occupational and Environmental Health, College of Public Health, University of Iowa, Iowa City, IA 52242, USA; kelly-k-baker@uiowa.edu; 2 Terre des Hommes, 1006 Lausanne, Switzerland; john.brogan@tdh.ch; 3 Department of Biostatistics, College of Public Health, University of Iowa, Iowa City, IA 52242, USA; daniel-sewell@uiowa.edu; 4 Terre des Hommes, Port-au-Prince 6111, Haiti; tdh.washgg.cdp@gmail.com; 5 Department of Civil and Environmental Engineering, Stanford University, Stanford, CA 94305, USA; lakwong@stanford.edu

**Keywords:** children’s health, environmental exposure, diarrheal disease, sanitation, solid waste, quantitative behavioral research, non-dietary ingestion, mouthing, public domains, Haiti

## Abstract

Globally, gastrointestinal (GI) infections by enteric pathogens are the second-leading cause of morbidity and mortality in children under five years of age (≤5 years). While GI pathogen exposure in households has been rigorously examined, there is little data about young children’s exposure in public domains. Moreover, public areas in low-income settings are often used for other waste disposal practices in addition to human feces, such as trash dumping in areas near households. If young children play in public domains, they might be exposed to interrelated and highly concentrated microbial, chemical, and physical hazards. This study performed structured observations at 36 public areas in an internally displaced persons community that has transitioned into a formal settlement in Haiti. We documented how often young children played in public areas and quantified behaviors that might lead to illness and injury. Children ≤5 years played at all public sites, which included infants who played at 47% of sites. Children touched and mouthed plastic, metal and glass trash, food and other objects from the ground, ate soil (geophagia) and drank surface water. They also touched latrines, animals, animal feces and open drainage canals. Hand-to-mouth contact was one of the most common behaviors observed and the rate of contact significantly differed among developmental stages (infants: 18/h, toddlers: 11/h and young children: 9/h), providing evidence that children could ingest trace amounts of animal/human feces on hands that may contain GI pathogens. These findings demonstrate that water, sanitation and hygiene interventions could be more effective if they consider exposure risks to feces in public domains. Furthermore, this research highlights the need for waste-related interventions to address the broader set of civil conditions that create unsafe, toxic and contaminated public environments where young children play.

## 1. Introduction

Globally, 1.7 billion episodes of diarrhea and around half a million diarrhea-related deaths occur in children under five years of age (≤5 years) living in low-to-middle income countries (LMICs) each year [1]. These gastrointestinal (GI) infections increase children’s risk of co-infection [2], environmental enteric dysfunction [3,4], growth faltering [5] and mortality [6]. Children are infected by GI pathogens through oral exposure to food, water, soil, objects and hands that have been contaminated with feces containing pathogens [7]. Water, sanitation and hygiene (WASH) interventions have aimed to reduce fecal-oral disease by targeting household practices such as water treatment and handwashing [8,9,10,11]. Rigorously evaluated household WASH interventions have yet to demonstrate significant, sustained reductions in children’s diarrheal disease [12,13,14]. Furthermore, the annual global incidence of diarrheal disease in children ≤5 years remains relatively unchanged after decades marking steady improvements in household water and sanitation access [11]. New global health intervention approaches are needed, which depend upon a better understanding of where and how young children are exposed to and infected by GI pathogens.

The persistent disease burden in children may be in part explained by exposures that occur in public rather than household areas—areas that would not be targeted by household WASH interventions. Community sanitation and hygiene conditions are known risk factors for disease in children [15,16,17]. While distinct mechanisms for fecal pathogen transmission through public and private exposure pathways were suggested decades ago [18], research on exposure risks has focused almost exclusively on household settings [8,19,20,21,22]. Research has established that young children are exposed to feces by placing contaminated objects and soil in their mouth in the household and yard [20,23,24,25]. Children’s hands are contaminated by feces when crawling, touching soil and objects on the ground [26], and touching animal feces [25]. Subsequent and frequent hand-to-mouth behaviors result in ingestion of fecal microbes and potentially pathogens, on the hands [21,26,27,28,29]. In addition, children can directly ingest pathogens by placing contaminated objects in their mouth or directly eating soil contaminated by pathogens [21,25,30,31]. Hand-to-mouth contacts in the household may result in even greater ingestion of feces than consuming unclean drinking water [20], indicating non-dietary ingestion during play may be an important pathway for diarrheal disease in young children. If children ≤5 years play in public areas that are impacted by open defecation, human feces disposal, deteriorated latrines, or animal feces, they could be exposed to elevated concentrations of a diverse range of GI pathogens in the environment. Exposure to these highly hazardous environments could cause GI infection in children, even when household WASH conditions are optimal. Understanding how often children ≤5 years play in public areas in LMICs and quantifying contact with objects that could lead to the ingestion of GI pathogens, is critical to informing policy stakeholders and public health practitioners about the potentially important role of public domains in child diarrheal disease.

For children that play in these public domains, diarrheal disease may not be the only waste-related risk impacting the health of children ≤5 years. Due to a lack of access to solid waste management services, open drainage canals and open lots are used for disposal of many forms of trash in addition to human feces [32,33,34]. Unmanaged trash dumping in neighborhood areas near households provides an opportunity for children to contact trash during play in public areas. Some literature has described the occupational health outcomes of rag- and waste-pickers including increased rates of infections [35,36,37]. However, these cohorts include children ≥8 years old who rummage through trash piles to find items that can be re-sold, reused, or recycled and may be unrepresentative of children ≤5 years who contact trash during play in public areas. Interestingly, one study found that dumping trash in the surrounding environment was the most important factor associated with diarrhea among children <2 years, after controlling for household sanitation conditions [38]. Transboundary movement of electronic waste (e-waste) into LMICs contributes to elevated chemical contamination of soil and air [39]. E-waste can leach several chemicals, including lead and cadmium that are especially toxic to women and children [39,40]. There is a need to better understand and quantify young child contact with trash during play in public areas, since these behaviors could result in exposure to feces-contaminated waste, chemicals, blood-borne pathogens and sharp objects [41]. Lastly, uncovered infrastructure in public areas such as open drainage canals [32] and latrine pits without a cover [42] pose immediate concerns for serious injuries, fatal falls and drownings if young children play in these areas.

Given the multitude of hazardous scenarios in public areas, our study aimed to describe how often young children are observed playing in public areas and to measure the rate at which children practice behaviors, such as hand and mouth contacts with objects that could lead to illness and injury. Quantifying the rate of interaction between young children and hazards in public areas is critical to understanding how current sanitation and waste practices impact the health and safety of children.

## 2. Methods

### 2.1. Community Context

Corail, Haiti, (est. pop. 14,500), originated as a planned camp for internally displaced persons (IDP) built on sparse, unused land 18 km northeast of Port-au-Prince following the earthquake in January 2010. Reflective of global humanitarian trends, Corail has transitioned to a permanent formal settlement of over 2500 families in the Croix des Bouquets commune, necessitating the need for development of permanent water and sanitation infrastructure. Corail’s provisional sanitation infrastructure (109 dry pit latrine blocks of four to six toilet cabins) was established as part of humanitarian relief efforts. Blocks of toilets were located on the periphery of rows of housing, with one toilet being shared by five to eight families. At the time of the study, most septage pits were full and in urgent need to be emptied and many latrines were in a state of disrepair. According to the residents of Corail, no routine waste management services were available and/or affordable to the community.

### 2.2. Study Design

The University of Iowa Institutional Review Board reviewed and approved this study (ID#201705786). Residents of the community were trained to perform structured observations of anonymous young children approx. ≤5 years playing at neighborhood public areas from unobtrusive locations. Our study design aimed to reduce bias that could occur if an observer followed a particular child. The behaviors of a child and caregivers’ interactions with their child could be modified when observers noticeably follow individual child participants for several hours [43]. Caregivers of child participants could feel obligated during scheduled study visits to keep their child within the parameters of the household for observer convenience, to conform to social expectations, or other unexplored reasons. Children may limit their behaviors and spatial range to engage with observers or to conform to what he/she understands to be acceptable behavior with the awareness of an observer following them. These behavioral modifications could reduce the typical time a young child plays outside of the household. Structured observations of unidentified children at fixed public locations allowed us to reduce observer bias and capture the behaviors of many different children visiting these public areas, at the cost of describing spatial movement patterns of individual children. 

### 2.3. Data Collection Tool

**Figure 1:**
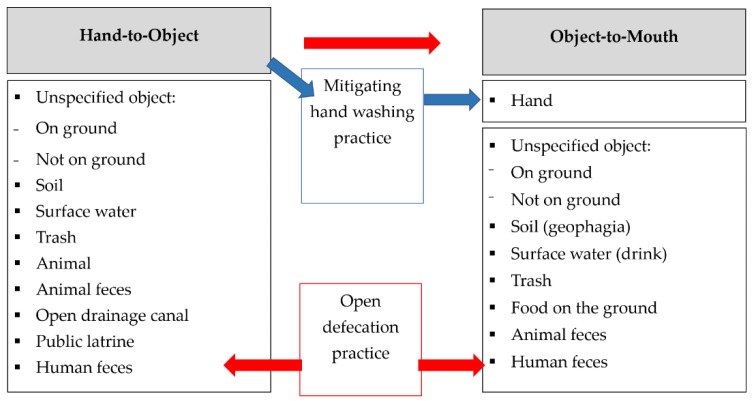
Conceptual framework for children’s contact with objects and hygiene and sanitation practices at public sites. Red arrows represent flow of feces into the environment and children’s mouths. Blue arrows represent behavior that would reduce feces transmission.

Our study aimed to document young children’s hand and mouth contact with objects and hygiene and sanitation practices listed in the a priori conceptual framework to understand exposure pathways that occur during play at public sites (Figure 1). Child open defecation practices contribute to contamination levels of the environment and increase other children’s risk of exposure to feces-contaminated surfaces. Hand contact with objects at public sites and the use of public latrines could result in the transfer of trace particles of feces containing pathogens onto the hand. Contact with sharp trash, such as broken glass or metal, poses direct risk for wound injury and infection of the hand. Handwashing diminishes contamination accumulated on the hands during prior contact with objects and is therefore reported as a mitigating behavior that could reduce or eliminate the transmission of contaminants on dirty hands placed in the mouth. Object-to-mouth contact at public sites such as geophagia (“earth eating”) could result in direct and heightened exposure to contaminants residing in the environment [25,44]. “Unspecified object” is defined as any object that did not fit the description of other objects. Unspecified objects were categorized by whether the object was on the ground prior to contact (i.e., rocks, leaves) or was not on the ground (i.e., walls, barriers, posts).

**Figure 2:**
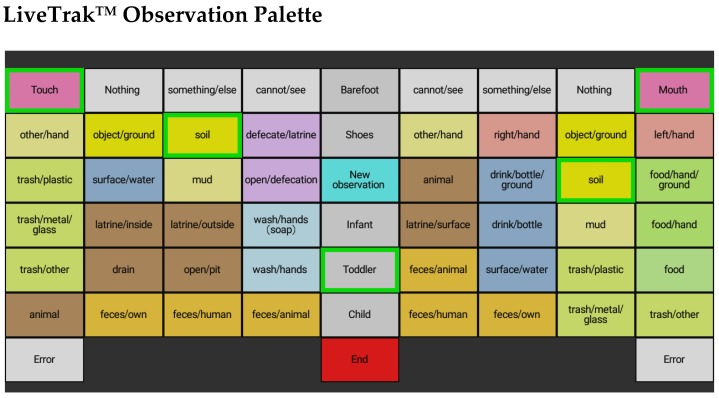
Customized LiveTrak™ application for public observations in Corail, Haiti (illustrated in English; a Kreyol version was used in the field). As depicted by the selected objects, the palette indicates that an observed toddler is touching soil and engaging in geophagia.

*Terre des hommes* staff, who are residents of Corail, participated in an intensive week-long training in structured observation of child behavior. LiveTrak™ software (Stanford University, Stanford, CA, USA) [21] was customized for observation of multiple anonymous children at public sites and their contact with a broad variety of waste stream materials, for example, plastics and metal (Figure 2). The tool was modified throughout the training process to improve the clarity of object classification and quality of information collected. The left half of the screen allowed observers to record objects touched by the child (e.g., soil, trash/plastic), while the right half of the screen allowed recording of objects that were placed in the child’s mouth (e.g., soil, hand). The center column allowed observers to begin a new observation, record whether the child was wearing shoes or was barefoot and record the approximate age of the child (infant, toddler, or young child). The application automatically timestamped each selection and sequentially recorded events in the form of a comma delimited (.csv) file.

### 2.4. Observation Training

We did not record the precise age of observed children because we did not enroll specific, identified children and their caregivers. We hypothesized that development of motor coordination and mobility, rather than age, is more relevant to child’s exposure to public areas beyond the domestic yard. Therefore, we classified children as infants, toddlers and young children up to approximately 5 years of age by developmental stages (Table 1).

Quantifying hand contact with objects requires defining how to record contacts that vary with frequency and duration. For example, a child could hold a ball in his/her hand for a prolonged duration of time (scenario 1), rapidly bounce a ball multiple times within a few seconds (scenario 2), or alternate between touching a ball and another toy (scenario 3). Each of these scenarios vary by the number of touch and release contacts with the ball and the duration of each contact. Attempting to record rapid hand contacts and track the duration of each of contact without the use of videography could result in imprecise measurement as observers become fatigued, especially over three-hour observation periods. Our study did not record prolonged contacts (scenario 1) or rapidly repetitive contacts (scenario 2) with the same object as multiple hand-to-object contacts. For example, if a child was sitting on the ground and playing in the soil, the observer would select “touch/soil” and keep the selection highlighted during repetitive contacts with the soil in the proximal area. If the child then touched an animal, the observer would select “touch/animal.” If the child returned to touching the soil, the observer would record a second occurrence of “touch/soil.” This approach results in lower counts of environmental contact with the same objects that could influence the microbial loading onto the hands from repeat contact with the same object. Yet, this approach focused on capturing important contacts with different objects that could result in new exposures to microbes. Our team agreed that recording every mouthing contact was possible with reasonable accuracy, since it was a less rapid behavior and thus recorded every object contact with the mouth. Similar to previous methods, observers recorded a contact even if the same portion of the object had previously entered the mouth [21,30].

During training, three observers watched and recorded the behaviors of children playing in various scenarios until they achieved an inter-observer Krippendorff’s alpha (α) agreement [46] of above 80%. When testing for inter-observer agreement in field-based training sessions, observers would identify and confirm with one another which child would be observed. Observers would then separate and remain within 5 m of one another and face the same direction to ensure a consistent line of vision while observing but refrain from communicating. Concordance remained high during several field sessions prior to data collection and throughout two comparison sessions during data collection (α ≥ 0.88).

### 2.5. Data Collection

Prior to data collection, the study team members reviewed research ethic guidelines and discussed procedures for addressing bystander inquiries. Observers were asked to intervene and stop a child from imminent harm if they witnessed a child performing a particularly risky behavior, such as placing glass in the mouth. Since the community members frequently interact with the children in the neighborhood through their involvement with the *Terre des hommes* child protection program, intervening to stop a child from harm was appropriate, expected and likely had minor influence on observation results since most interactions were brief verbal warnings. We did not record how many times observers intervened to stop a child from performing a behavior for concern that the additional task to record communication with children would interrupt observers’ focus to continuously record behaviors.

Observation sites were chosen from 83 randomly selected locations that were previously tested for enteric pathogen presence for a public domain exposure project (unpublished). Sites were classified into two sub-groups: residential sites with no public latrines present (“residential sites”) and sites on the perimeter of the neighborhood within 15 m or less of a shared latrine (“perimeter-latrine sites”). We selected for a 2:1 ratio of residential to perimeter-latrine sites, respectively, to examine child-play patterns across the two sub-settings. Our study had a target goal of at least 30 observation sites. A total of 64 sites were pre-selected in the event that time allowed for more data collection. Observation periods were rotated between mornings (starting between 8–9 a.m.) and afternoons (starting between 3–4 p.m.) when the sun was less intense and children were more likely to be outside of the household. Morning and afternoon sessions were not randomly pre-determined since this was primarily dependent upon what the daily agenda allowed. The three trained observers were individually assigned different perimeter-latrine and residential sites during morning and afternoon sessions with minimal partiality.

**Figure 3:**
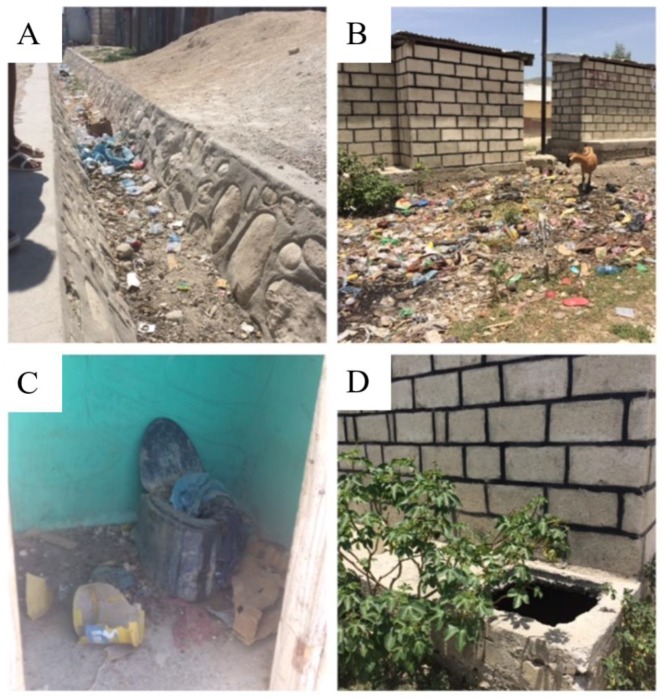
Illustration of environmental conditions in public areas. (**A**) Open drainage canal used for trash disposal; (**B**) Goat roaming in a trash pile behind a latrine; (**C**) Public latrine in poor condition; (**D**) Latrine pit without a cover.

Once observers arrived at the designated site and found a suitable location to observe, real-time GPS coordinates were recorded using KoboToolbox™ (Harvard Humanitarian Initiative, Cambridge, MA, USA). Using a paper survey, environmental conditions were documented based upon the presence of latrine structures, animals, feces (human/animal) and the types and locations of trash (e.g., in a latrine pit, open drain, pile behind latrine) (Figure 3). Human and animal feces were identified by visual inspection and observers were trained to identify distinguishing features using scatology and signs of human feces disposal (plastic bags, chamber pot dumping, etc.). After assessing the site for environmental conditions, observers spent a duration of three consecutive hours recording behaviors of children who visited and played at the public site. If an observed child left the site or no children were playing at the site, the observer would select “cannot see (a child)” and would scan the area for other children who were approximately five years or younger. If a child remained at a site and was observed for 30 min, the observer would re-scan the area for other children to observe. If other children were present, the observer would transition to a new observation, otherwise the observer would continue to record the behaviors of the child playing at the site. This method allowed us to capture behaviors of many different children of all developmental stages. Due to unpredictability in the number of children playing at public sites and the possibility that children could exhibit large ranges of spatial movement, we were not able to track whether children already observed returned to the site and were observed during a later occurrence. Data collected from each site was reviewed with observers and any additional observations/comments were discussed among the research team each day after observations.

### 2.6. Data Analysis

Qualitative descriptions of public sites were summarized using the environmental survey completed prior to the beginning of observation according to the presence of latrine structures, open drainage canals, human feces, animals and their feces and various trash dumping locations at public sites. Observation data were cleaned and contact behaviors per child observation were summed using Microsoft Excel 2016 (Redmond, WA, USA). Krippendorff’s alpha inter-observer agreement and all data analyses were performed using RStudio version 3.4.1 (Boston, MA, USA). The total time children were present at a public site was calculated by subtracting all time when “cannot see (a child)” was selected from the total observation time (about three hours per observation period). A generalized linear model based on the Gamma distribution with an identity link was used to examine whether total time when children were present at public sites varied by time of day (morning/afternoon) and location (residential vs. perimeter-latrine sites). A generalized linear model based on a Bernoulli distribution with a logit link was used to examine whether wearing shoes was influenced by site location (residential vs. perimeter-latrine sites) and developmental stages.

The number of sites where at least one child was observed to engage in a behavior (Figure 1) was reported as “site-level exposure.” The number of individual child observations within each developmental stage who engaged in a behavior at any site was reported as “child-level exposure.” For child-level exposure, it is important to note that transition to a new observation was frequent due to the rapid pace of children moving in and out of the proximal area during play and the large number of children interacting at each site. Thus, the likelihood of observing an individual child display a specific behavior over a short duration was limited, which is why site-level exposure is helpful in understanding the overall diversity of contact with hazards at public sites.

We modeled the contact counts for each of the 20 behaviors according to one of four probability distributions described below using the sum of contacts per individual child observation and the respective duration of each observation. The developmental stage of the child (infant/toddler/child) was included in the models except for cases in which one or more contact events were recorded for less than five child observations within each developmental stage. In such cases, we pooled the data across all developmental stages. First, we fit the data to a Poisson distribution with the time of the child observation as an offset and when applicable the developmental stage as a covariate. Second, we fit the data to a negative binomial distribution where again the time of the child observation was used as an offset, the developmental stage as a covariate (when applicable), as well as a dispersion parameter, thereby allowing higher variances of the count data. Our third and fourth probability distributions fit to the data were zero-inflated variants of the same Poisson (ZIP) and negative binomial distributions (ZINB). The ZIP assumes that there is a sub-population that does not engage in the behavior while the remaining segment of the population engages in the behavior according to a Poisson distribution; it is similar for the ZINB. 

After fitting the data to each of the four distributions, we constructed a quantile-quantile plot (QQ plots) using a Monte Carlo approximation of the hypothesized distribution conditioning on the actual counts of child behavior and using parameter values estimated from the data. If the hypothesized distribution is correct, we expect to see a QQ plot with all points close to the line with intercept of zero and slope of one. For each behavior, we selected the distribution that seemed most appropriate according to the QQ plots. In cases where two or more distributions fit the data equally well, we chose the simplest of the models (e.g., Poisson is simpler than a ZIP).

Identifying the distribution of the data can provide insight into the underlying practice of behaviors among children in a population. If the counts corresponding to a behavior follow a Poisson, then within each developmental stage a child has approximately the same rate of contacts. If the counts follow a negative binomial, this implies a greater degree of heterogeneity among children’s rates. This can be seen more clearly by noting that the negative binomial random variable is equivalent to a Poisson random variable whose rate is a random draw from a gamma distribution. That is, if a behavior follows a negative binomial distribution, each child’s rate of behavior is a random draw from a gamma distribution with rate and shape parameters both equal to the dispersion parameter from the negative binomial regression model. If a behavior is best modeled via a zero-inflated distribution, this implies that there is a subset of the population which does not engage in the behavior and the remainder of the population’s behavior frequencies follow a Poisson or a negative binomial distribution, as the case may be.

## 3. Results

### 3.1. Environmental Conditions

Observations were conducted at 36 sites, of which 18 were residential sites and 18 were perimeter-latrine sites (Figure S1). Perimeter-latrine sites were more likely to contain open drainage canals, human feces and obvious piles of trash (Table 2). Regardless of location, plastic and metal trash was present at all sites, while glass trash was observed at 86% of all sites. E-waste was present at 33% of all sites. Trash was scattered around the region at practically every site (97%). Open drainage canals always contained trash (Figure 3). Three perimeter-latrine sites had trash piles, suggesting that these areas were community-designated locations for trash dumping (Figure 3). Although it was not always possible to confirm presence of trash inside of latrine pits, at least 50% of latrine pits contained trash. Prior to observation, the presence of human feces in the surrounding environment was recorded at eight perimeter-latrine sites and one residential site. As a result of children practicing open defecation during the time of observation, four additional sites, including two residential sites, were reclassified as containing human feces. In total, 36% of sites were contaminated with human feces. Animal feces were observed at every site. Goats, dogs and cats were observed most often. Domestic birds such as chickens and ducks were seen at about 80% of sites and pigs were present at less than half of sites.

### 3.2. Frequency of Child Play at Public Sites

Three-hour observations at the 36 sites were conducted, totaling more than 100 h of public observation. Children estimated to be ≤5 years were present for an average of 1 h 40 min of the total three-hour public observation period per site (55% of total observation time). After accounting for transition time between observations, 51% (range 14–98%) of observation time was committed to observing child behaviors, resulting in 54.8 h of child observation. The duration of time when children were observed playing did not significantly vary between the morning and afternoon observation sessions (*p* = 0.73). The duration of time when children were observed playing at perimeter-latrine sites was significantly less by approximately 26 min compared to residential sites (*p* = 0.02).

We recorded a total of 386 observations of children (240 young children, 117 toddlers and 29 infants) and the average time spent observing each child was approximately 4.8 min (geometric mean) and ranged from 15 s to 80 min. See Figure S2 for the distribution of time spent observing each child. A young child (between toddler and ≤5 years) was observed at all 36 sites, toddlers at 92% of sites and infants at 47% of sites. At least one young child, toddler and infant was observed at 100%, 94% and 44% of perimeter-latrines sites (n = 18), respectively. Approximately 41% of children were barefoot, which significantly differed between infants (60%), toddlers (52%) and young children (30%) after adjusting for site location (*p* < 0.001). Children were more likely to be wearing shoes at perimeter-latrine sites compared to residential sites, after adjusting for developmental stages (*p* = 0.03).

Children were observed touching and mouthing a diverse range of objects at public sites, including soil, trash, surface water, animals, animal feces, open drainage canals and ate food that was observed to be on the ground prior to ingestion. These behaviors occurred in residential and perimeter-latrine sites and across various proportions of total time when children were present (Figure S1). The number of sites and the number of children within each developmental stage observed to engage in a behavior are reported in Table 3. The selected models that most appropriately fit the distribution of respective behaviors (Figures S3–S22) and estimated contact rates are reported in Table 4.

### 3.3. Hand-to-Object Contacts

Children of all developmental stages touched non-trash objects on the ground at all 36 sites. As described by observers, commonly touched objects on the ground (not specified) were toys, rocks and leaves. Using a Poisson model, the rate of touching objects on the ground differed across developmental stages (*p* < 0.001). Pairwise tests revealed significantly higher rates of touching objects on the ground for young children (27 contacts/h) versus toddlers (19/h) but not toddlers versus infants (20/h). Children touched metal/glass trash at the majority of all sites (75%), although this behavior was observed for less than 15% of all children. Using a ZIP model, the pooled probability of children touching metal/glass trash is 22% and when children do engage in this behavior, the rate of contact is 9/h. Site- and child-level exposure to plastic/other trash was more frequent than contact with metal/glass trash. As such, children have an increased probability of touching plastic/other trash (38%) compared to touching metal/glass trash. 

Children of all developmental stages touched soil at all sites. The rate of touching soil differed across developmental stages (*p* < 0.001). Using a ZIP model, the probability of infants, toddlers and young children touching soil is 58%, 87% and 69%, respectively. When children touch soil, infants have a significantly higher rate (15/h) than toddlers (10/h) and young children have a significantly higher rate (16/h) than toddlers. At least one child in each developmental stage touched surface water at seven sites, although the behavior was infrequent. Using a negative binomial (NB) model, the pooled rate for touching surface water was less than one contact/hour. 

Toddlers and young children touched open drainage canals at 40% of 20 sites where drains were present. Observers described young children entering drainage canals to retrieve toys such as a soccer ball. Using a ZIP model and only including observations at sites with open drainage canals, the pooled probability of children contacting an open drainage canal is 13% and when they do engage in this behavior, the contact rate is 5/h. Children touched animals at 36% of sites, although only one infant was observed to contact an animal. Using a ZIP model, the pooled probability of children touching an animal is 11% and when children do engage in this behavior, the rate of contact is 4/h. Toddlers and young children touched animal feces at five sites, while no infants touched animal feces. Observers described children using goat feces as an alternative for marbles to play a popular children’s game.

### 3.4. Handwashing Practices

Young children and toddlers washed their hands without soap at five public sites. No children washed their hands with soap at public sites. Handwashing was not observed at critical times including after defecation or prior to eating. It is unclear what prompted handwashing in most cases; one child washed his/her hands after touching surface water and two children washed their hands after touching plastic trash. No handwashing stations were present at public sites. Observers described children typically washing their hands with water from a sachet of water provided by their caregiver. Using a NB model, the estimated pooled rate of handwashing without soap was infrequent at 0.17 events/hour.

### 3.5. Object-to-Mouth Contacts

Children of all developmental stages frequently placed their hands in their mouths at all sites. Using a Poisson model, the rate of hand-to-mouth contact differed across developmental stages (*p* < 0.001). Pairwise tests revealed significantly higher rates of hand-to-mouth contact for infants (18/h) versus toddlers (11/h) and significantly higher rates for toddlers versus young children (8/h). Using a NB model, the rate of mouthing objects on the ground differed across developmental stages (*p* = 0.03). Pairwise tests revealed significantly higher rates of mouthing objects on the ground only for infants (4/h) versus young children (1/h). Mouthing metal/glass trash was observed at 22% of sites, was infrequent for toddlers and young children (<5%) and never observed among infants. Children of all developmental stages mouthed plastic/other trash at 42% of sites, although this behavior was observed for less than 15% of all children. As such, children have an increased probability of mouthing plastic/other trash (17%) compared to mouthing metal/glass trash (4%) using the ZIP model.

Ingestion of soil occurred most frequently among infants and was observed at six sites. Using a ZIP model, the pooled probability of children engaging in geophagia is 16% and when children do engage in this behavior, the rate of contact is one/h. A total of three children (one toddler and two young children) drank directly from surface water at two sites. The estimated pooled rate was less than one drinking occurrence/hour using a NB model. One surface water source was described as laundry water, while the other source was unknown. Children of all developmental stages ate food from the ground at 39% of sites with a pooled estimated rate of less than one per hour using a NB model.

### 3.6. Sanitation Practices

At 72% of perimeter-latrine sites, nineteen young children and four toddlers used a public latrine both alone or with the assistance of others, although frequency of supervision was not recorded in this study. Using a Poisson model and including only perimeter-latrine sites (n = 18), the estimated pooled rate for using a public latrine was about one occurrence/hour. Observers recorded children contacting the interior walls and surfaces of the latrines before/after using the toilet. However, precise measurement of contact with latrine surfaces was not recorded out of respect for children’s privacy during use of the facility. Two young children and four toddlers practiced open defecation at six sites. Four of these events occurred at perimeter-latrine sites, while the other two events occurred at residential sites, indicating children are not limited to practicing open defecation exclusively in areas bordering the neighborhood. One instance of a young child touching a latrine pit without a cover was observed.

### 3.7. Contact Trends across Time Spent Playing at Public Sites

**Figure 4:**
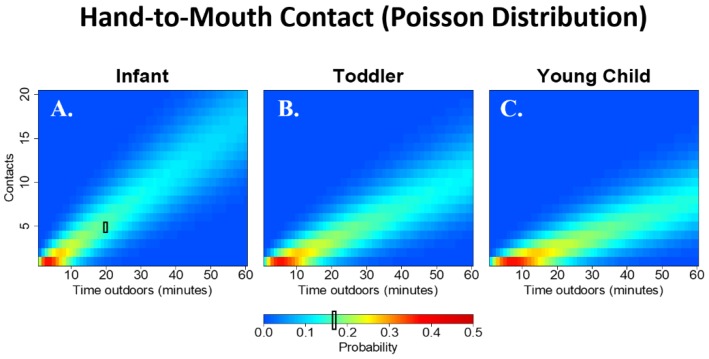
Temporal trends using the probability density of a Poisson distribution parameterized by the lambda of hand-to-mouth contacts for infants (**A**); toddlers (**B**); and young children (**C**). Each vertical slice represents the probability of observing a discrete number of contacts given that the child plays in a public area for a specific time-span. For example, the probability of an infant engaging in 5 hand-to-mouth contacts during 20 min of play at a public site is about 16% (black box in **A**).

**Figure 5:**
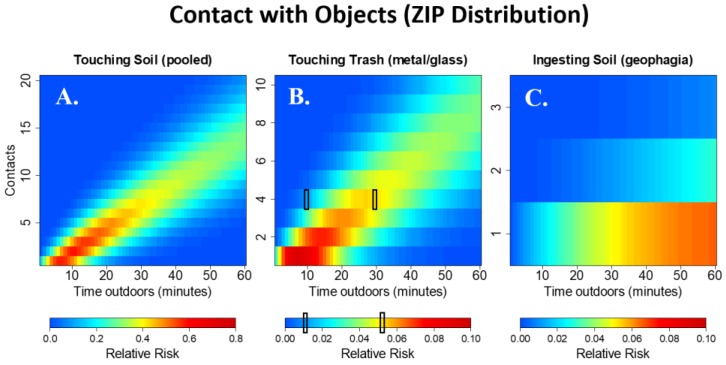
Temporal trends using the relative risk of engaging in one or more contacts compared to no contacts, as estimated from fitting the zero-inflated Poisson distribution. Each vertical slice represents the relative risk of a child engaging in a certain number of contacts (y-axis) compared to no contacts during play in a public area for a specific time-span (x-axis). For example, the probability of a child touching trash 4 times is less than 0.02 times the probability of never touching trash during 10 min of play at a public area (far-left black box in **B**). During 30 min of play at a public area, the probability of touching trash 4 times increases to more than 0.05 times the probability of never touching trash (far-right black box in **B**). Note the scale adjustment for the relative risk when comparing across figures.

Figure 4 shows the probability distribution for the number of hand-to-mouth contacts among infants (A), toddlers (B) and young children (C) while playing in public areas for 1 to 60 min; that is, each vertical slice provides the probability of engaging in a discrete number of observed contacts given that the child plays in a public area for a specific time-span. The number of expected hand-to-mouth contacts decreases with age. Figure 4 also illustrates how exposure may increase with more time spent playing in public areas due to more total counts in relationship to more total time spent playing in public areas. Figure 5 shows the relative risk (rather than probabilities for purposes of visual clarity) of engaging in one or more contacts compared to not engaging in the behavior at all using a ZIP distribution. For example, the probability of having one contact with soil during ten minutes of play in a public area is 0.5 times the probability of having zero contacts during the same time interval. Note that while individual cell values may be low, the overall relative risk of seeing one or more contact events may be high. For example, the relative risk of one or more contacts with soil during 30 min of play in a public area versus no contacts during the same time interval is 2.78. The relative risk of one or more hand contacts with soil (Figure 5A) is much greater than the relative risk of engaging in touching trash (Figure 5B) and geophagia (Figure 5C). When children touch metal/glass trash, they are likely to engage in more contacts compared to geophagia, where it is rare to observe more than one contact.

## 4. Discussion

This study found that infants, toddlers and young children living in a LMIC setting play in neighborhood public areas and contact hazards that pose potential microbial, chemical and physical health risks. This study quantified the rate of contact between young children (≤5 years) and a diverse range of waste-related hazards, including trash and physical hazards during play in public areas. Children often touched soil and objects and placed soil, objects and hands in their mouth, which could lead to the ingestion of trace amounts of observed human and animal feces potentially contaminated by enteric pathogens that could cause GI infection [47]. Significantly more hand-to-object contacts, including touching objects on the ground and soil, among young children compared to infants and toddlers suggests children with increased mobility might be exposed to more contaminated fomites. Yet, higher rates of hand-to-mouth contact among infants and toddlers could lead to substantially greater ingestion of trace contaminants even if they contact fewer fomites. Observed contact with trash, drains and latrine pits add to these risks, highlighting a concern for opportunistic wound infections, injury and dermal-based routes of exposure to chemical and hazardous waste. Children were often barefoot and significantly higher rates of barefooted play among infants and toddlers suggests the youngest children are more vulnerable to hookworm and to injury of the feet that could result in infection. Greater amounts of time spent playing in public areas might increase young children’s exposure to enteric pathogens and other contaminants via more total hand and mouth contacts with objects. Contact with soil and trash was common compared to other measured behaviors and could result in repeat oral ingestion of fecal pathogens [47,48,49,50]. Hand contact with latrines, drains, animals and animal feces were infrequent, yet these items may transmit high amounts of feces or waste contaminants. Infrequent observation of geophagia indicates that direct ingestion of contaminants in soil is less common, yet even occasional exposure in public areas could lead to the ingestion of high concentrations of microbes/contaminants. Evidence of child exposure in neighborhood public play areas contaminated by human and animal feces and other waste streams suggests that public play areas might contribute to diarrheal disease burden, as well as other waste-related diseases among children ≤5 years. A sister study in a peri-urban settlement in Kenya determined soil in public areas with observed feces contamination was frequently contaminated by many different types of pathogens and also confirmed young children played at these public sites [47]. A forthcoming manuscript will integrate environmental pathogen data collected in Corail (using a similar approach described in Baker et al., 2018 [47]) with our study’s behavioral data to estimate young children’s exposure to enteric pathogens during play in public areas.

Identifying the distribution of contact rates for measured behaviors provided insight into the underlying practices among children playing in public areas, given our short-term observations. Common behaviors such as touching objects on and off the ground and hand-to-mouth contact fit a Poisson distribution, indicating all children are likely to engage in these behaviors and within each development stage, children have a similar rate of contact. Although touching soil was a common behavior among children, the ZIP model was selected because it was a slightly improved fit compared to the Poisson model. Using the ZIP model, the majority of children in all developmental stages engaged in touching soil. Eating food from the ground and mouthing objects not on the ground fit a NB model, indicating a greater degree of heterogeneity among children’s rate. Most of the less frequent behaviors such as touching trash, open drainage canals and geophagia fit a ZIP model, indicating there is a subset of the child population that do not engage in these behaviors over the course of observation time-spans recorded in this study. Children may not engage in certain behaviors during play in public areas for reasons including a lack interest in or reason to contact some objects (e.g., no intention to enter an open drainage canal if a toy did not fall into the drain) or negative prior experiences resulting from contact with some objects (e.g., previous injury from broken glass). Rates of very infrequent behaviors such as drinking surface water and open defecation were estimated with the simplest model (Poisson or NB). While the ZIP/ZNB model might seem more theoretically sound since not all children are likely to practice open defecation or drink surface water, the limited data did not provide enough information to estimate the probability of engaging in the behavior. Thus, we opted for the simpler model. Behavioral data was reported per protocol despite limited information about some behaviors that were infrequent. Less frequent behaviors inherently increase uncertainty about the underlying distribution and results, yet not reporting these behaviors would exclude potentially high-risk exposures. Researchers who use these results in modeling or seek to replicate study protocol should scrutinize the uncertainty of the data and consider whether the reported results for especially infrequent behaviors are of sufficient quality for their study.

In this study, hand-to-object contact was recorded when children contacted objects that could result in exposure to potentially contaminated objects. We did not document rapidly repetitive contacts with the same object as multiple contacts due to concern that observers would struggle to both pay attention to the child and reliably record every single behavior, especially over three-hour observation periods. Other studies have used videography to record fine-scale hand contact with objects and have included detailed information about left/right hand involvement and repetitive/constant contact [26,28] but we were unable to take this approach because videography was not appropriate to implement in Corail. Our reported rates of hand-to-object contact episodes are lower than the individual contact rates reported for children in previous videography studies because we did not have the capacity to record fine-scale hand contacts that can be examined with time-delayed review of videography [26,28]. Additionally, differences in contact rates among previous literature and our study could in part be explained by variation in observation setting, the types of objects available to children during observation, how objects are defined, categorized and reported in studies and the statistical modeling methods used to estimate rates. 

Rates of hand-to-mouth contact per hour are consistent with previous studies of child behaviors in outdoor settings in high-income countries, including videography observations [27,31]. However, Kwong et al. (2016) reported a hand-to-mouth rate of 34/h for children 6 to 12 months in household yards [21], which is higher than our estimates and those of previous studies [27,31]. Discrepancies may be explained by Kwong et al. recording hand-to-mouth contacts while infants were eating. When pooling all non-dietary object-to-mouth contacts, we found that our study estimates using a Poisson model (infants: 7.2/h, toddlers: 4.7/h and young children: 3.5/h) are reasonably comparable to reported outdoor object-to-mouth rates from other literature [27,28,51,52].

Estimated contact rates for other behaviors such as touching soil and geophagia are difficult to compare to previous literature because of the additional parameter used in the ZIP model to estimate the probability of a child engaging in the behavior, which has not been considered in other studies. However, when children do engage in touching soil our contact rate is comparable to rates reported by Ko et al. (2006) [53] and Reed et al. (1999) [54]. Our estimated geophagia rate of 0.9/h for children who engage in the behavior falls between Kwong et al. (2016) estimates for soil ingestion ranging from 0.1–0.3/h for children 3 to 18 months [21] and Ngure et al. (2013) estimates ranging from 1.7–2/h for children 3 to 18 months [25].

Observation of unidentified children who enter public areas is useful to reduce the bias that may arise from observing select children whose caregivers may be present and attempt to modify the child’s behavior. Alternatively, public observations could introduce community reactivity bias if caregivers call their children into the home if they do not feel comfortable with their child being observed. Community concerns were addressed by explaining the purpose of the study and ensuring only the trained community members and no researchers were present during data collection. Our observation method was not able to estimate the time individual children play in public areas (versus domestic areas) over the course of a day because we did not follow individual children. Further information about the spatial pattern of young children and time spent outside of the household and yard is needed to determine the cumulative daily exposure to enteric pathogens for a child. The models used to estimate the rate of behaviors allows for flexibility in adjusting the duration of exposure when more information about individual children play patterns outside domestic areas are better understood.

Many risks faced by children in our observation were caused by sanitation and waste practices that stemmed from inadequate waste management services in Corail. Children played at sites on the perimeter of the neighborhood that contained deteriorating latrines with open septage pits—areas that were also used for open defecation, feces and trash disposal. Deteriorating latrine infrastructure increased the likelihood of communities utilizing these same locations for waste dumping, essentially creating hazardous hotspots that pose interrelated potential microbial, chemical (e-waste) and physical injury risks to children. In addition, children in this setting must travel farther from home to reach these perimeter-latrine sites and the notable lack of adult supervision at public sites amplifies the concern for children playing unattended in unsafe and unsanitary environments. Young children’s unsupervised use of latrines and lack of handwashing pose several health and safety concerns. Default sanitation practices, such as child open defecation, that occur from lack of functional toilets and could be influenced by caregiver supervision contribute to the presence of human feces in public areas. This study did not record whether observed children were supervised. Supervision of children became an obvious and important consideration after the study tool was implemented since the majority of young children played in public areas unattended during observations. Therefore, it would be useful for future research to quantify the level of supervision to describe how supervision influences children’s behaviors in public areas. 

The peri-urban community of Corail is reflective of global relief trends: a former camp transitioning to a permanent settlement with over 2500 families lacking permanent water and sanitation infrastructure at the time of the study. However, there are limitations to generalizing information obtained in this study to other LMIC settings since the waste and sanitation conditions of Corail are likely extreme given the recent and emergency circumstances that led to the development of the community. Specifics such as latrine conditions and location and density of trash and drainage canals may not reflect the landscape of other communities and could influence the rate at which children contact certain hazards. Yet, lack of sanitation and trash management services continue to be a prevailing issue in LMIC settings, especially for informal settlements due to voluntary or involuntary displacement and environmental conditions within such communities that are likely to be impacted by waste-dumping practices. Thus, child exposure to trash and feces outside domestic domains is likely a widespread public health issue in LMIC communities with varying degrees depending upon the unique characteristics of each community.

Practical interventions that integrate civil, social and environmental aspects will be required to address health and safety issues described in this study. Low rates of handwashing with water or soap among young children at public sites suggests that practices that would stop the transmission of pathogens by dirty hands are rare. Even if handwashing was practiced often, the frequent rate of contact with objects would quickly negate the benefits of handwashing. Thus, an intervention targeting handwashing practices is unlikely to be effective in reducing children’s exposures during play in public areas. In crowded settlements like Corail, where household space is limited, it is equally unrealistic to try to prevent children from playing in public sites. However, play in dangerous public settings and unsupervised use of public latrines and open defecation practices might be mitigated with the promotion of caregiver supervision and education. Provision of sustainable safe sanitation alternatives that can be used by young children could reduce contamination of public areas. Critically, increasing latrine coverage and eliminating play around areas with heightened risks (i.e., latrine structures) might reduce human feces exposure risks for children but will not eliminate the multitude of other pervasive trash and animal feces hazards at public sites. The results of this study provide a strong case for investing in interventions that improve neighborhood hygiene conditions outside of the household, especially those that end the perpetual cycle of waste and feces dumping in public areas. Further—the current fragile state of Corail serves as a reminder that such communities, formed as a result of displacement, often have a weak social fabric and absence of essential public services. To prevent such dire living conditions for families who are already vulnerable during times of crisis, aid relief and international policies must stress the importance of linking short-term relief efforts with long-term development programs to provide sustainable services for victims of natural and other disasters. 

## 5. Conclusions

This study documents that children ≤5 years, including infants and toddlers, play in neighborhood areas outside the home and display behaviors that lead to non-dietary ingestion of feces-impacted soil and objects that could contain enteric pathogens. This reveals that current WASH interventions targeting fecal exposures inside the household are not addressing all (and potentially critical) pathways that lead to GI infections in young children. The lack of attention to exposures that occur when young children play in unsanitary and unsafe public areas could in part explain why rigorously evaluated WASH interventions have yet to prove sustained, significant impact on diarrheal disease in children [12,13,14]. To create substantial improvements in decreasing diarrhea morbidity rates among young children, it may be beneficial to integrate interventions that target pathways outside the household in addition to classic WASH approaches. The stark diversity of environmental hazards contacted during play at public sites reveal implementation of interventions tackling only feces exposures in public areas would fail to embrace the opportunity to address other dangerous exposures that impede the health and safety of children. Thus, a new holistic public health approach should be adopted which addresses the broader set of civil conditions that create unsafe, toxic and contaminated public environments where children play.

## Figures and Tables

**Table 1:** Developmental stage characteristics used by observers to classify sub-groups of children.

Infant	Toddler	Young Child
Crawl or sit	Early walker	Walks well
Can stand only with assistance from props	Unsteady running gait	Plays with older children but has limited ability to keep up with games
	Large head-to-body ratio *	

* Head is less than one-fourth the total body length (infant) but greater than one-seventh the total body length (adult); approx. one-fifth the total body length [45].

**Table 2:** Proportion of observation sites with environmental conditions indicated.

	Perimeter-Latrine Sites n = 18	Residential Sites n = 18	All Sites n = 36
Open drainage canals	78% (14)	33% (6)	56% (20)
Human feces *	56% (10)	17% (3)	36% (13)
Animal feces	100% (18)	100% (18)	100% (36)
Animals	Goat: 88% (16)	Goat: 100% (18)	Goat: 94% (34)
	Dog/Cat: 94% (17)	Dog/Cat: 100% (18)	Dog/Cat: 97% (35)
	Pig: 39% (7)	Pig: 22% (4)	Pig: 31% (11)
	Poultry: 89% (16)	Poultry: 72% (13)	Poultry: 81% (29)
Any trash	100% (18)	100% (18)	100% (18)
E-waste	39% (7)	28% (5)	33% (12)
Trash scattered	100% (18)	94% (17)	97% (35)
Trash in a drain	78% (14)	33% (6)	56% (20)
Trash in a pile	17% (3)	0% (0)	8% (3)
Trash inside latrine pit	50% (9)		

* Includes human feces presence as a result of child open defecation during observation.

**Table 3:** Number of observation sites (site-level exposure) and children (child-level exposure) observed to practice a behavior.

	Site-Level Exposure n = 36	Child-Level Exposure (n = 386)		
		Infants n = 29	Toddlers n = 117	Young Children n = 240
**Hand-to-Object**				
Unspecified object:				
- On ground	100% (36)	79% (23)	74% (86)	68% (164)
- Not on ground	97% (35)	83% (24)	76% (89)	73% (175)
Soil	100% (36)	45% (13)	54% (63)	45% (107)
Surface water	19% (7)	3% (1)	3% (3)	2% (4)
Trash (metal/glass)	75% (27)	3% (1)	9% (10)	15% (35)
Trash (plastic/other)	83% (30)	17% (5)	12% (14)	22% (53)
Animal	36% (13)	3% (1)	4% (5)	4% (10)
Animal feces	14% (5)	0% (0)	2% (2)	2% (4)
Open drainage canal	40% (8/20) *	0% (0)	3% (4)	4% (10)
**Hygiene Practice**				
Wash hands (without soap)	17% (6)	0% (0)	2% (2)	2% (5)
**Object-to-Mouth**				
Hand	100% (36)	83% (24)	55% (64)	47% (112)
Unspecified object:				
- On ground	67% (24)	31% (9)	20% (23)	10% (25)
- Not on ground	53% (19)	10% (3)	9% (11)	6% (15)
Soil (geophagia)	17% (6)	10% (3)	2% (2)	1% (2)
Surface Water (drink)	5% (2)	0% (0)	1% (1)	1% (2)
Trash (metal/glass)	22% (8)	0% (0)	3% (3)	2% (5)
Trash (plastic/other)	42% (15)	10% (3)	7% (8)	4% (9)
Eating food on the ground	39% (14)	14% (4)	3% (4)	4% (10)
**Sanitation Practices**				
Use of a public latrine	72% (13/18) †	0% (0)	3% (4)	8% (19)
Open defecation	17% (6)	0% (0)	3% (4)	1% (2)

* Of the 20 sites where an open drainage canal was present; † Of the 18 perimeter-latrine sites.

**Table 4:** Estimated rates of hand and mouth contact with environmental hazards and related hygiene and sanitation practices.

	Model	Behavior/h (95% CI)		Dispersion (NB)/Probability of Engaging (ZIP)	
**Hand-to-Object**					
Unspecified object:					
- On ground	P ***	Infant: 20.20 (16.80, 24.03)	‡	N/A	
		Toddler: 19.24 (17.28, 21.35)			
		Child: 26.61 (24.84, 28.47)			
- Not on ground	P ***	Infant: 11.62 (9.09, 14.58)	†	N/A	
		Toddler: 18.74 (16.80, 20.82)			
		Child: 18.13 (16.67, 19.67)			
Soil	ZIP ***	Infant: 15.36 (11.81, 19.97)	†‡	Infant PE: 0.58	†
		Toddler: 10.45 (8.77, 12.45)		Toddler PE: 0.87	
		Child: 16.20 (14.50, 18.11)		Child PE: 0.69	
Surface water	NB	0.27 (0.11, 0.64)		Disp: 0.0325	
Trash (metal/glass)	ZIP	8.99 (7.19, 11.24)		PE: 0.22	
Trash (plastic/other)	ZIP	8.31 (6.91, 9.99)		PE: 0.38	
Animal	ZIP	4.43 (2.50, 7.82)		PE: 0.11	
Animal feces	ZIP	1.18 (0.31, 4.45)		PE: 0.11	
Open drainage canal +	ZIP	4.98 (2.63, 9.44)		PE: 0.13	
**Hygiene Practice**					
Wash hand (no soap)	NB	0.17 (0.07, 0.37)		Disp: 0.0719	
**Object-to-Mouth**					
Hand	P ***	Infant: 17.51 (14.35, 21.09)	†‡	N/A	
		Toddler: 11.16 (9.68, 12.79)			
		Child: 8.48 (7.50, 9.55)			
Unspecified object:					
- On ground	NB *	Infant: 4.37 (2.02, 10.43)		Disp: 0.3093	
		Toddler: 2.56 (1.55, 4.24)			
		Child: 1.49 (1.00, 2.20)			
- Not on ground	ZIP	3.58 (2.10, 6.12)		PE: 0.22	
Soil (geophagia)	ZIP	0.91 (0.14, 5.70)		PE: 0.16	
Surface water (drink)	NB	0.15 (0.04, 0.81)		Disp: 0.0069	
Trash (metal/glass)	ZIP	7.67 (4.03, 14.60)		PE: 0.04	
Trash (plastic/other)	ZIP	3.93 (2.49, 6.20)		PE: 0.17	
Eat food on the ground	NB	0.60 (0.34, 1.08)		Disp: 0.0796	
**Sanitation Practices**					
Public latrine use ++	P	1.06 (0.70, 1.53)		N/A	
Open defecate	P	0.11 (0.04, 0.22)		N/A	

P: Poisson, NB: Negative binomial; ZIP: Zero inflated Poisson; Disp: Dispersion (NB only), PE: Probability of engaging in behavior (ZIP only); Analyses restricted to sites with an + open drainage canal or a ++ public latrine; Overall significant rate differences between developmental stages at * *p* < 0.05, *** *p* < 0.001; † Significant difference between infant and toddler (*p* < 0.01); ‡ Significant difference between toddler and young child (*p* < 0.01).

## Supplementary Materials

The following are available online at http://www.mdpi.com/1660-4601/15/8/1646/s1, Figure S1: Map of observation sites in Corail (n = 36), Figure S2: Distribution of time spent observing each child at public sites (n = 386), Figure S3:Hand to Object: Unspecified object not on the ground, Figure S4: Hand to Object: Unspecified object not on the ground, Figure S5: Hand to Object: Soil, Figure S6: Hand to Object: Surface water, Figure S7: Hand to Object: Trash (metal/glass), Figure S8: Hand to Object: Trash (plastic/other), Figure S9: Hand to Object: Animal, Figure S10: Hand to Object: Animal feces, Figure S11: Hand to Object: Open drainage canal, Figure S12: Hygiene practice: Wash hands (without soap), Figure S13: Object to mouth: Hand, Figure S14: Object to mouth: Unspecified object on the ground, Figure S15: Object to mouth: Unspecified object not on the ground, Figure S16: Object to mouth: Soil (geophagia), Figure S17: Object to mouth: Surface water (drink), Figure S18: Object to mouth: Trash (metal/glass), Figure S19: Object to mouth: Trash (plastic/other), Figure S20: Object to mouth: Eating food on the ground, Figure S21: Object to mouth: Use of a public latrine, Figure S22: Sanitation practices: Open defecate.
