# Supplementary material for: Where Children Play: Young Child Exposure to Environmental Hazards during Play in Public Areas in a Transitioning Internally Displaced Persons Community in Haiti
Source: Int J Environ Res Public Health. 2018 Aug 3;15(8):1646. doi: 10.3390/ijerph15081646 (PMC6122025; doi:10.3390/ijerph15081646)

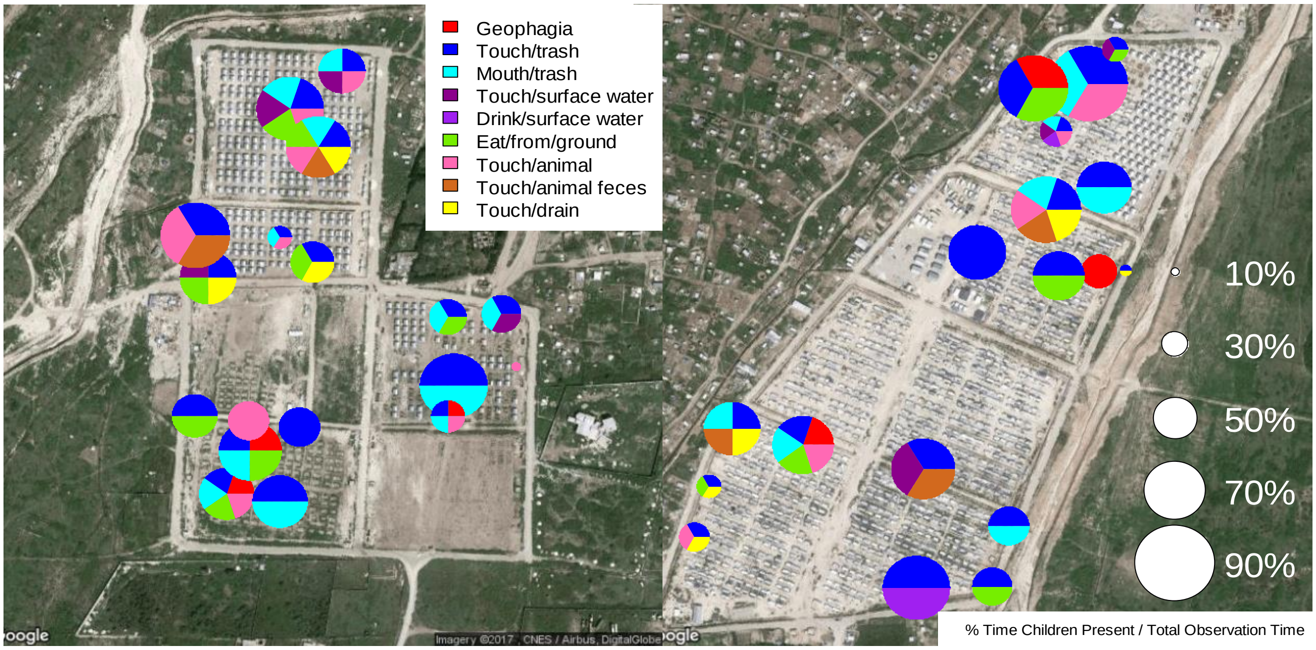

Figure S1. Map of observation sites in Corail (n=36). Occurrence of touching and mouthing contacts with diverse environmental hazards at observed public sites. Circle circumference expressed as the percent of total time children were present.

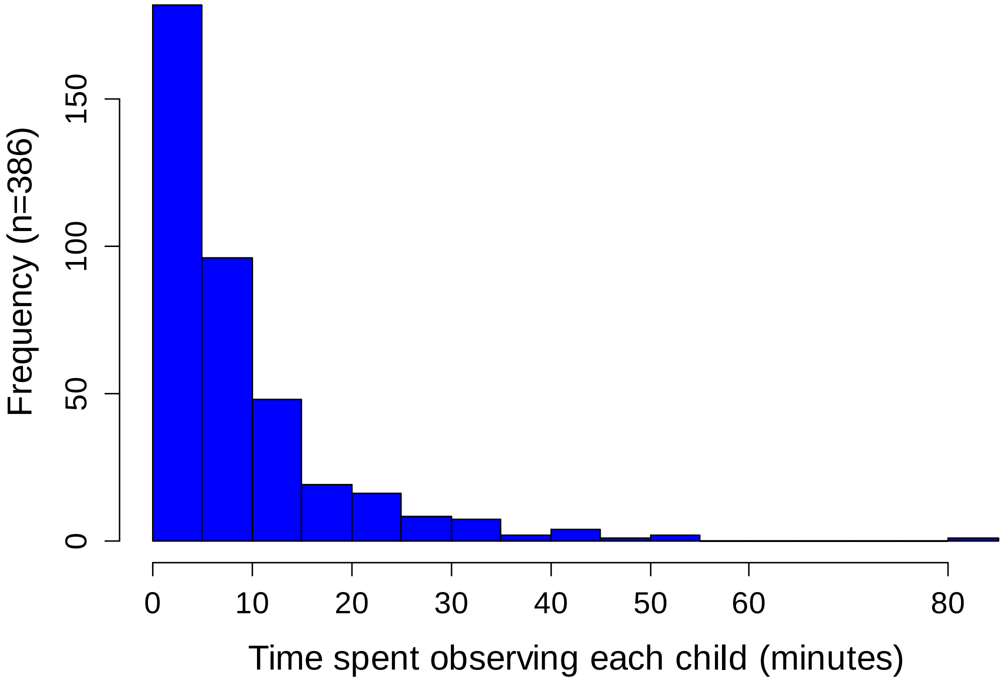

Figure S2. Distribution of time spent observing each child at public sites (n= 386).

Figure S3. Hand-to-Object: Unspecified object not on the ground

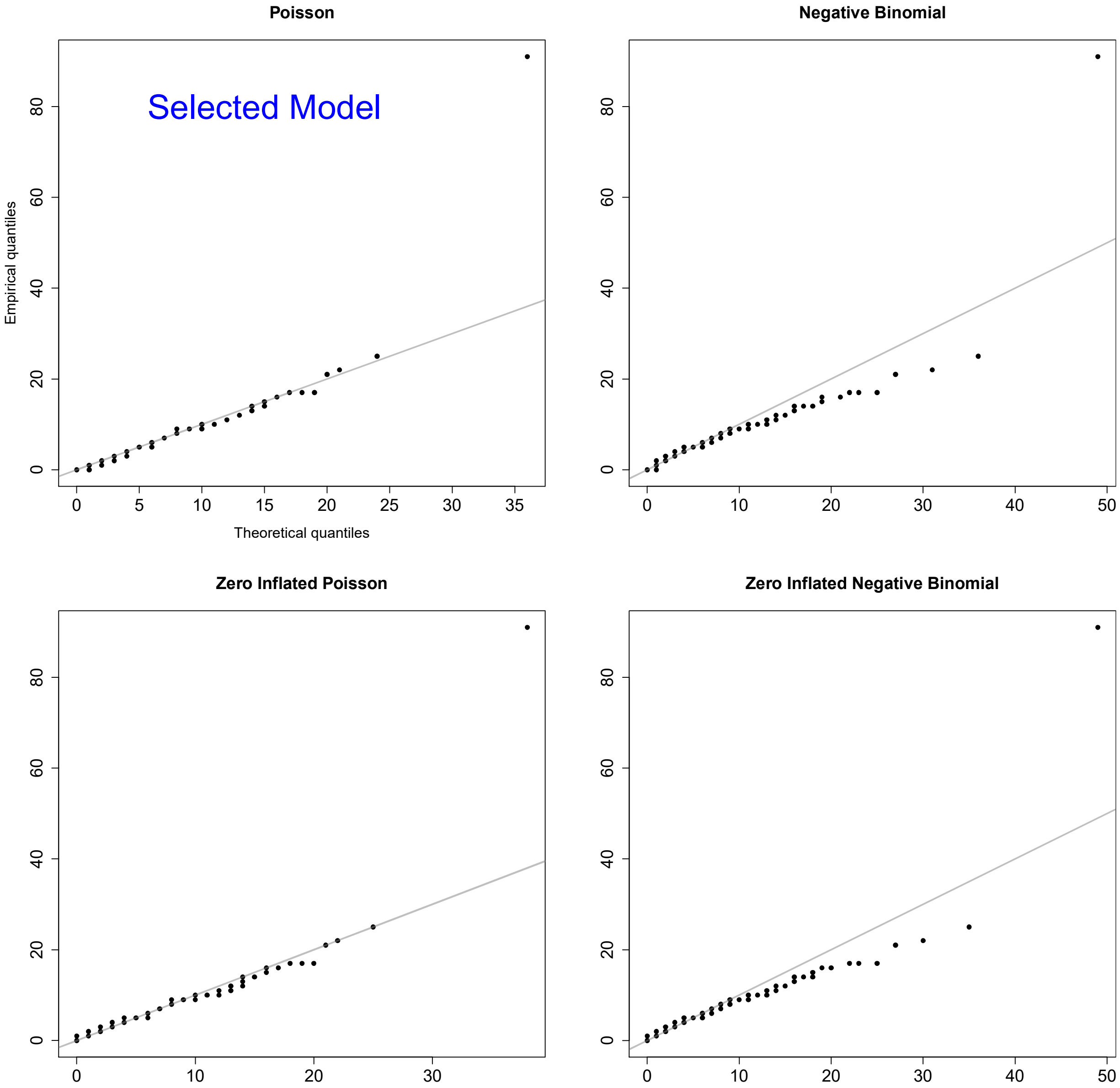

Figure S3-S22: Quantile-quantile (QQ) plots of hypothesized distributions used to estimate the rate of twenty different behaviors: Poisson (top-left), negative binomial (top-right), zero-inflated Poisson (bottom-left), and zero-inflated negative binomial (bottom-right). QQ plots were constructed using a Monte Carlo simulation of the hypothesized distribution conditioning on the actual counts of child behavior and respective model parameters estimated from the data. The best fit model is visually selected by determining which quantile points of a hypothesized distribution closely follow the line with intercept zero and slope of one.

Figure S4. Hand-to-Object: Unspecified object not on the ground

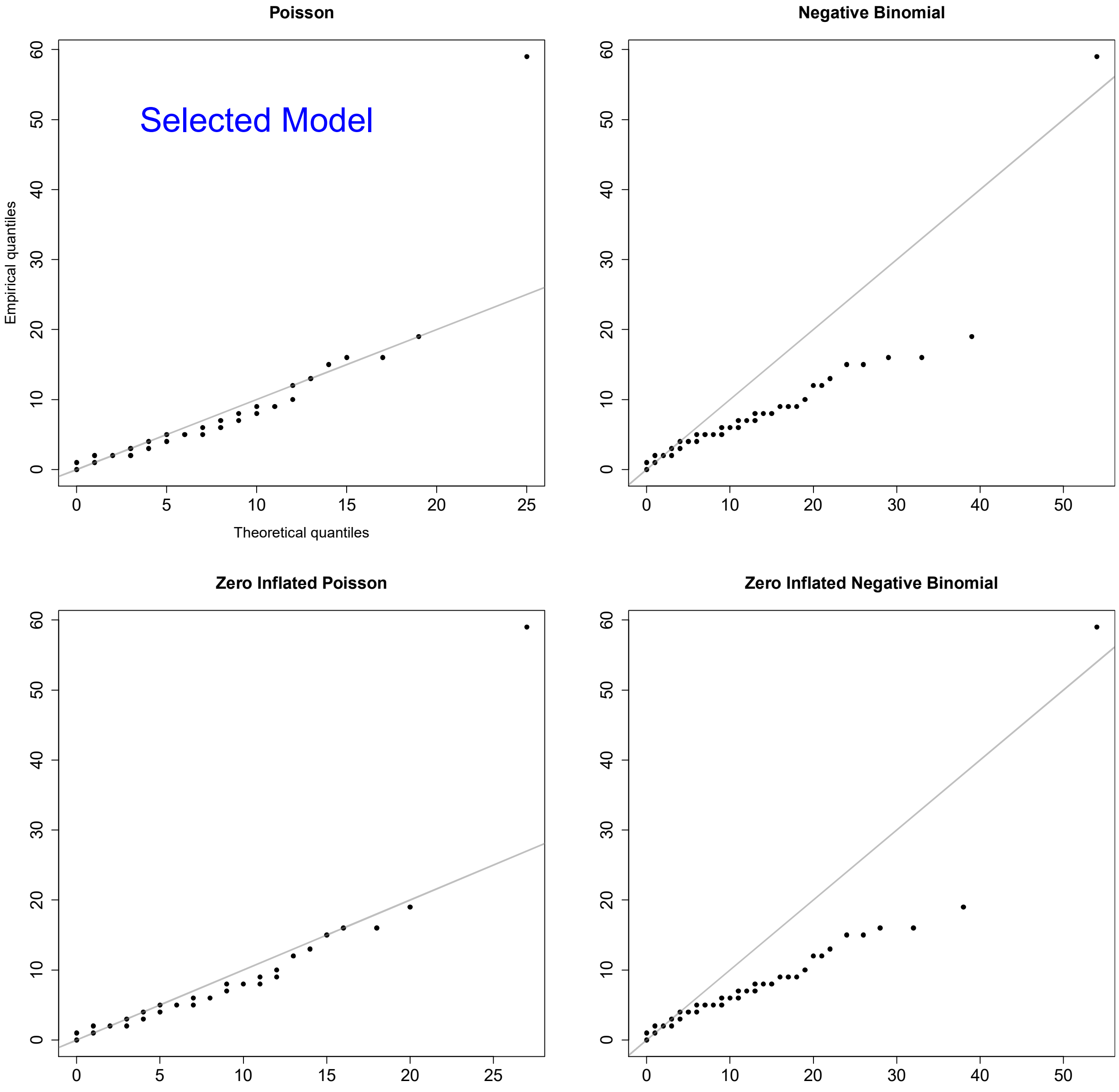

Figure S5. Hand-to-Object: Soil

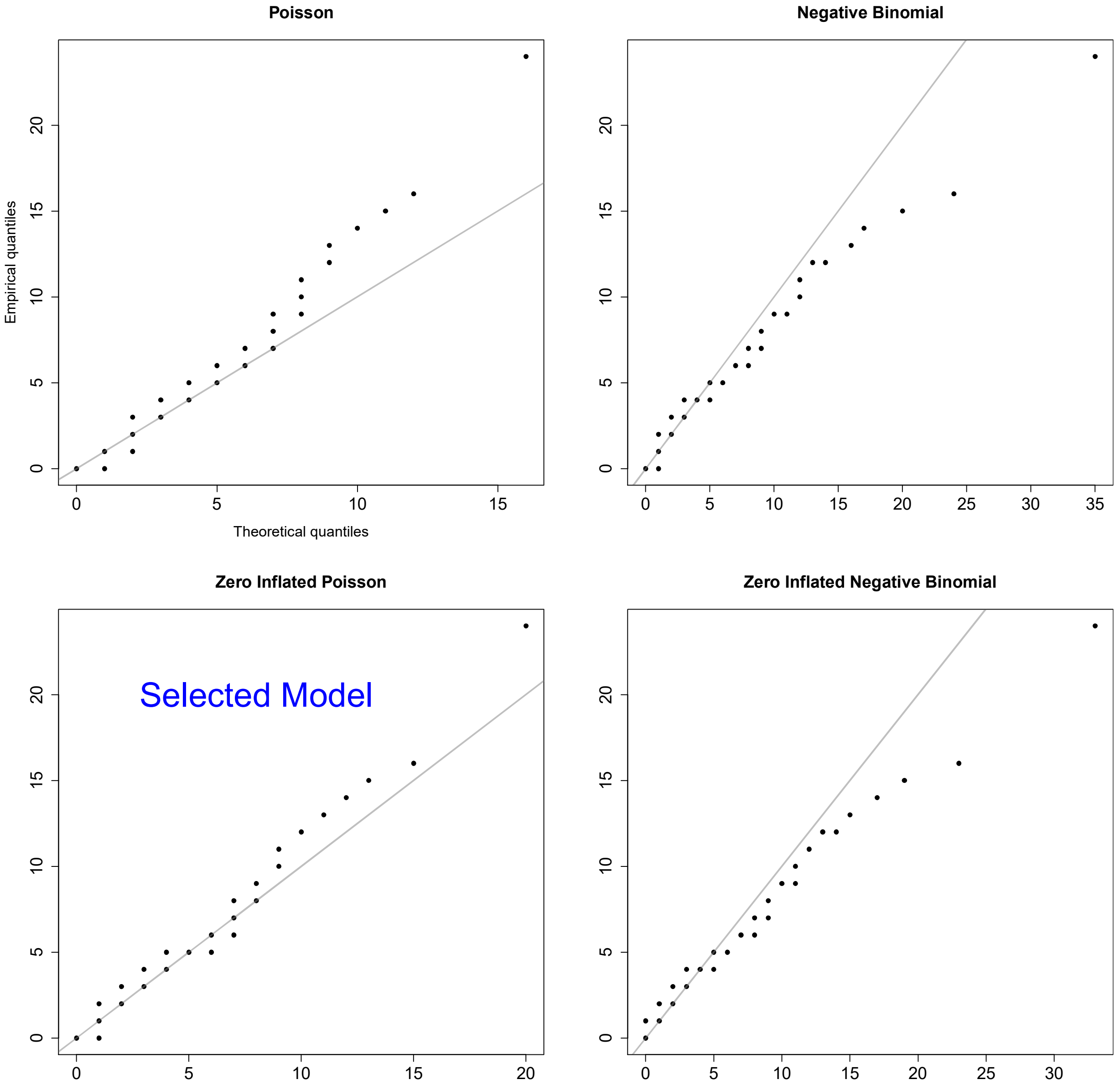

Figure S6. Hand-to-Object: Surface water

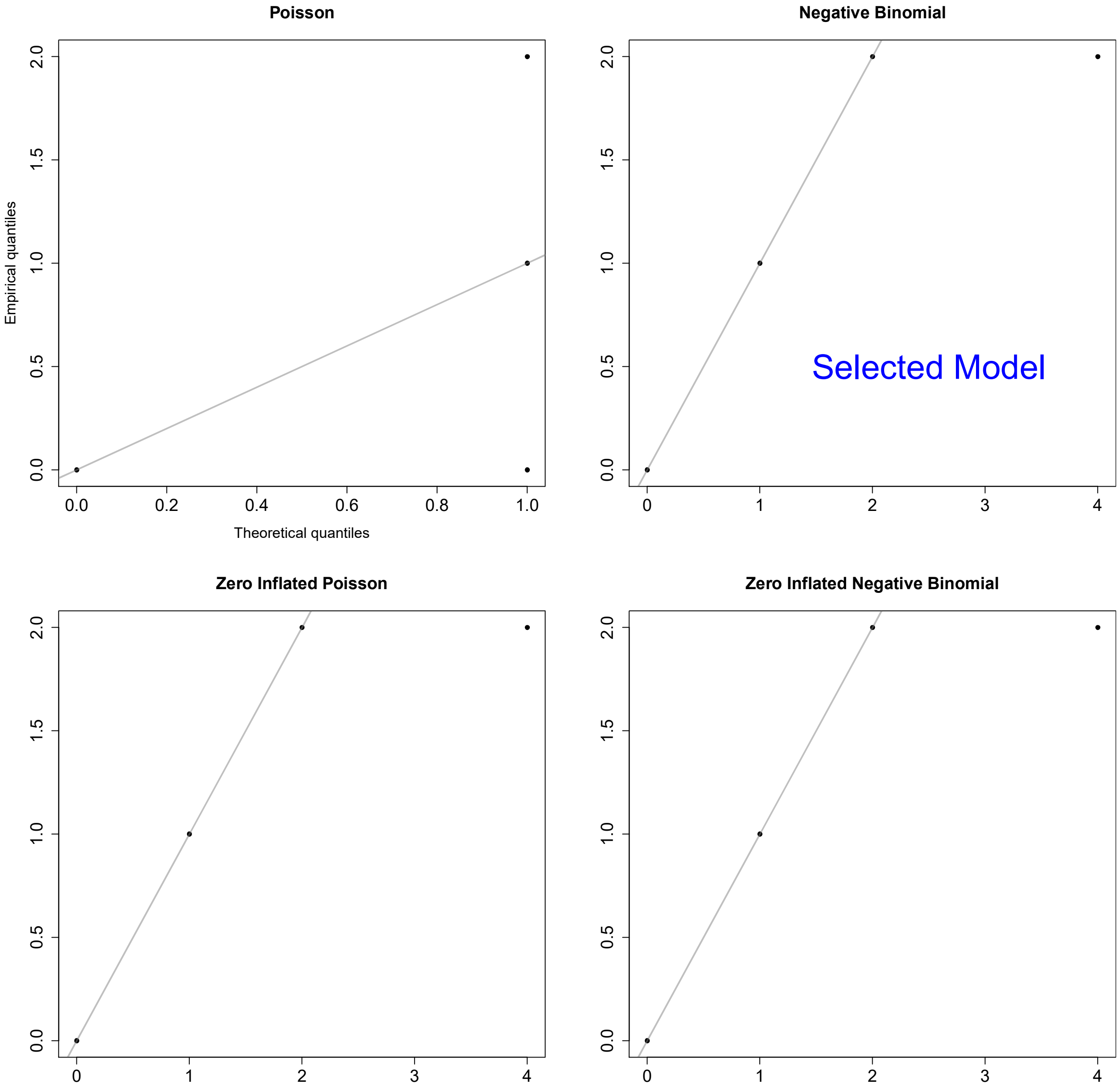

Figure S7. Hand-to-Object: Trash (metal/glass)

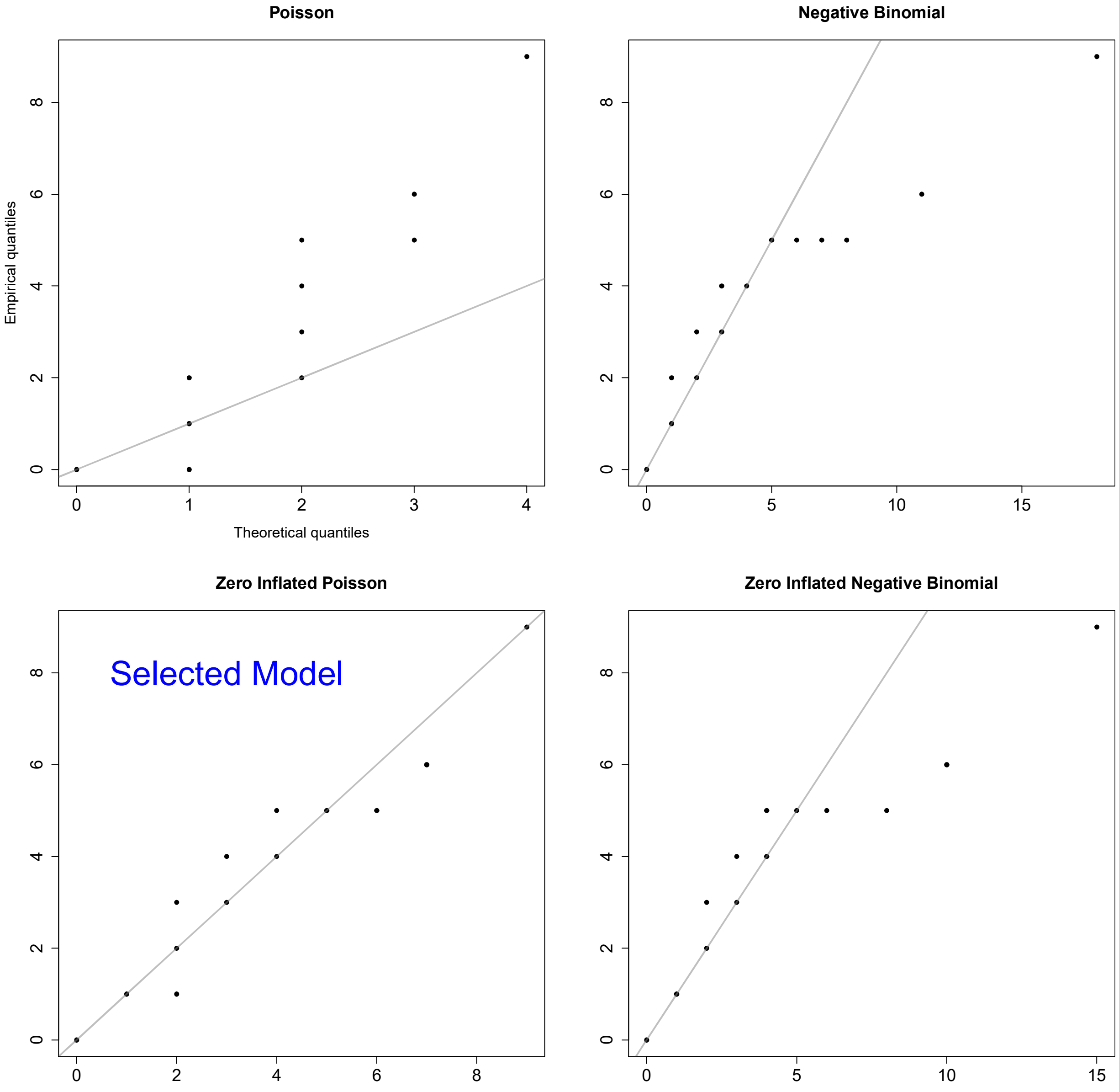

Figure S8. Hand-to-Object: Trash (plastic/other)

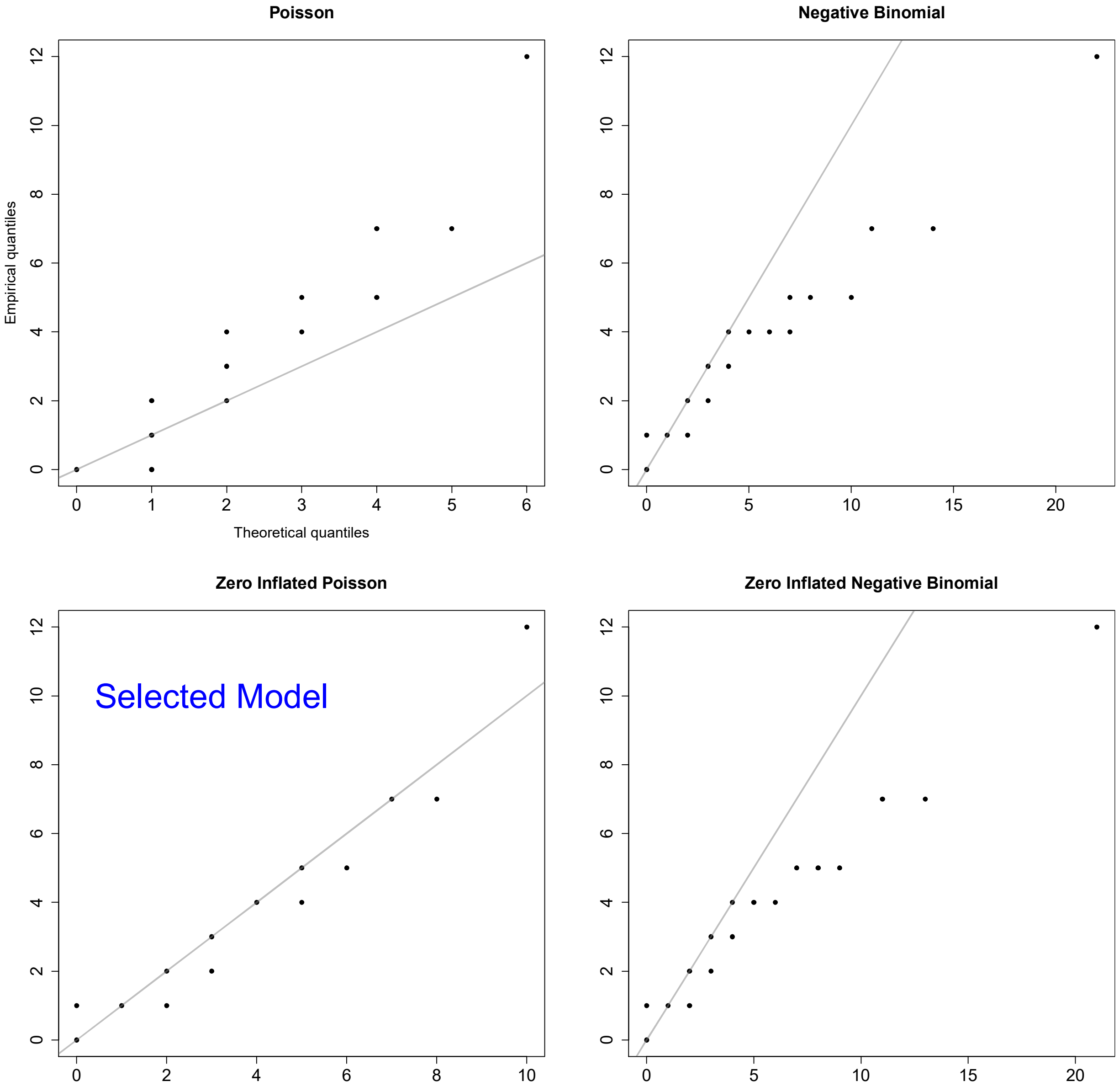

Figure S9. Hand-to-Object: Animal

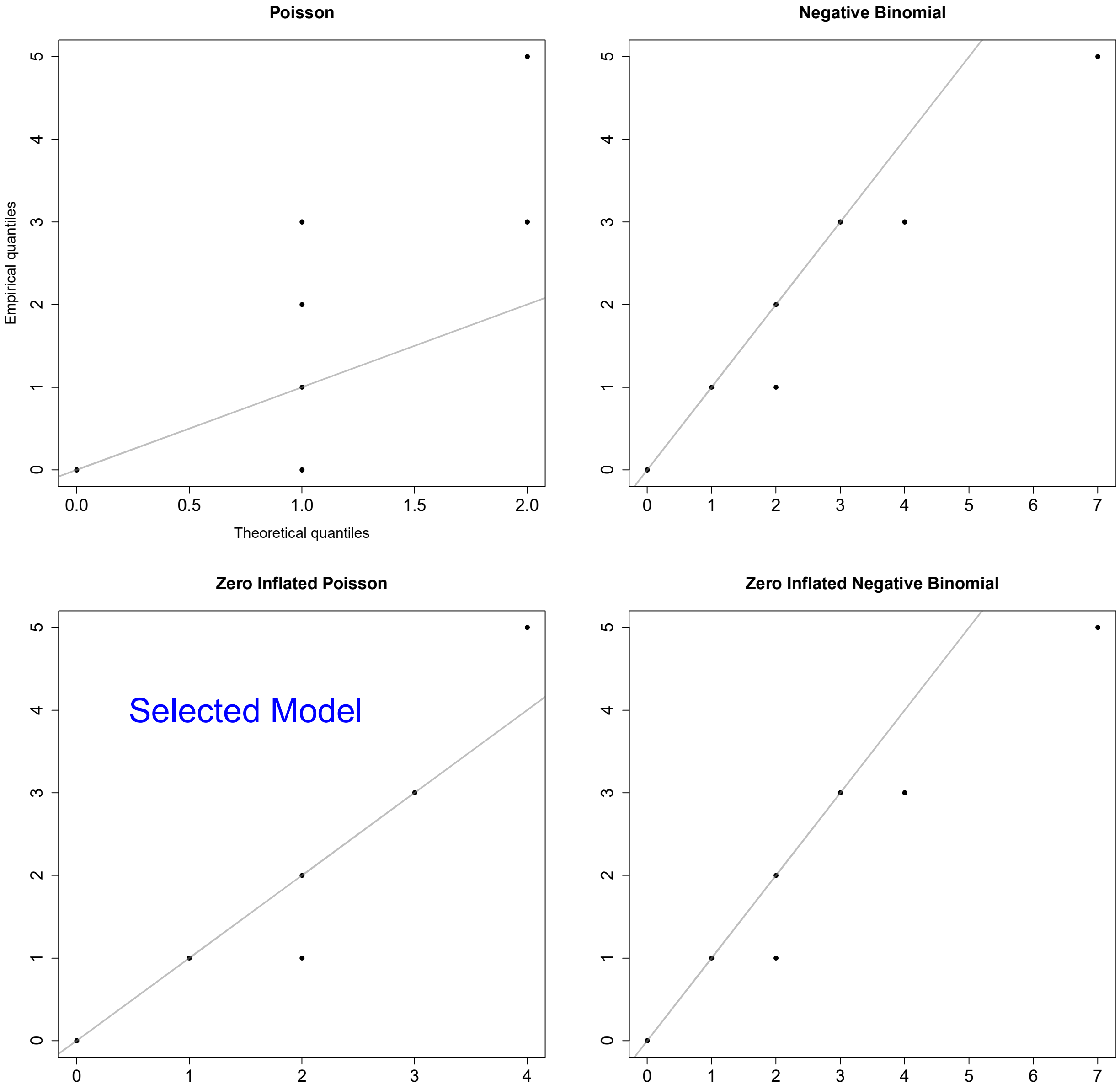

Figure S10. Hand-to-Object: Animal feces

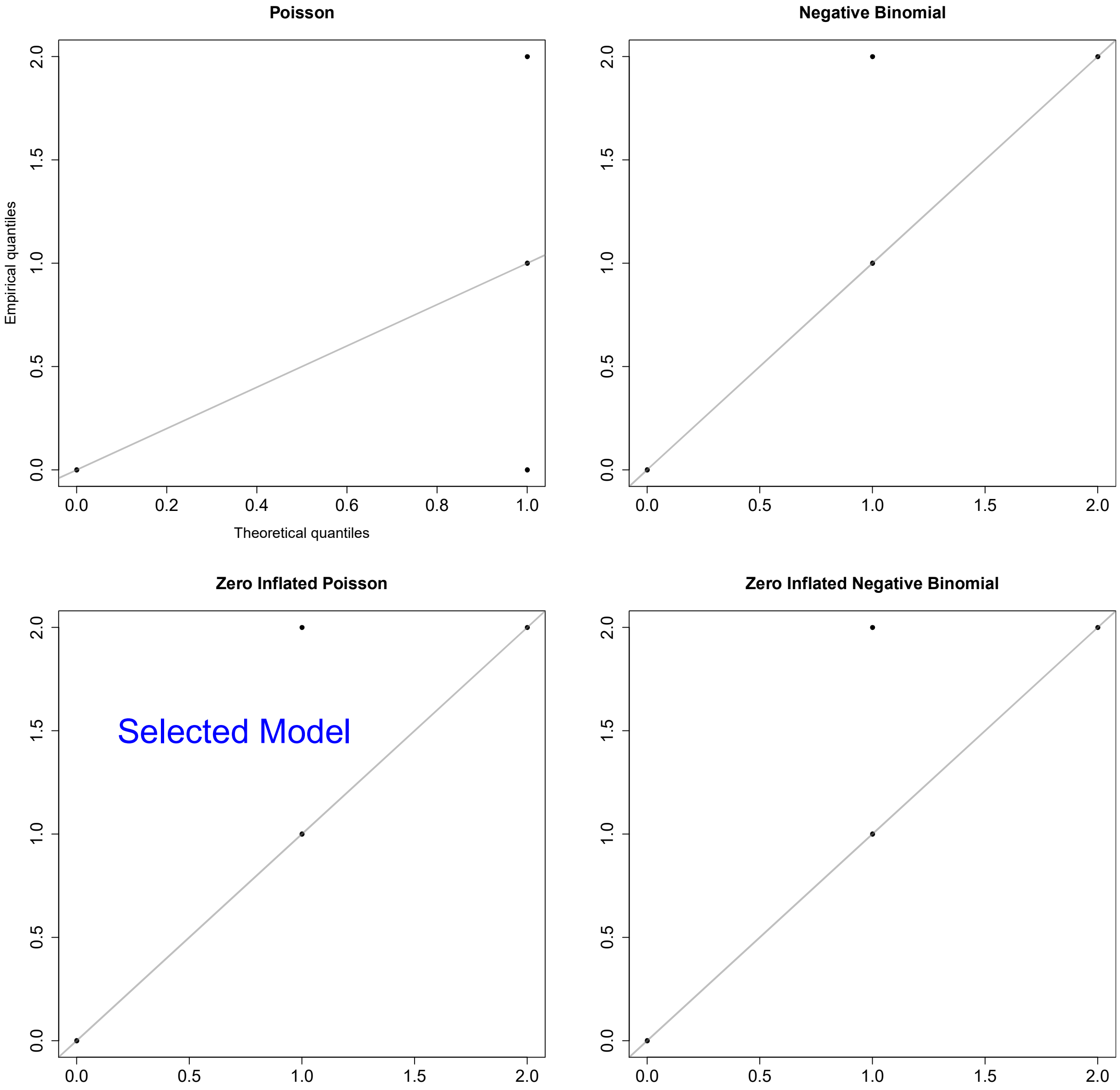

Figure S11. Hand-to-Object: Open drainage canal

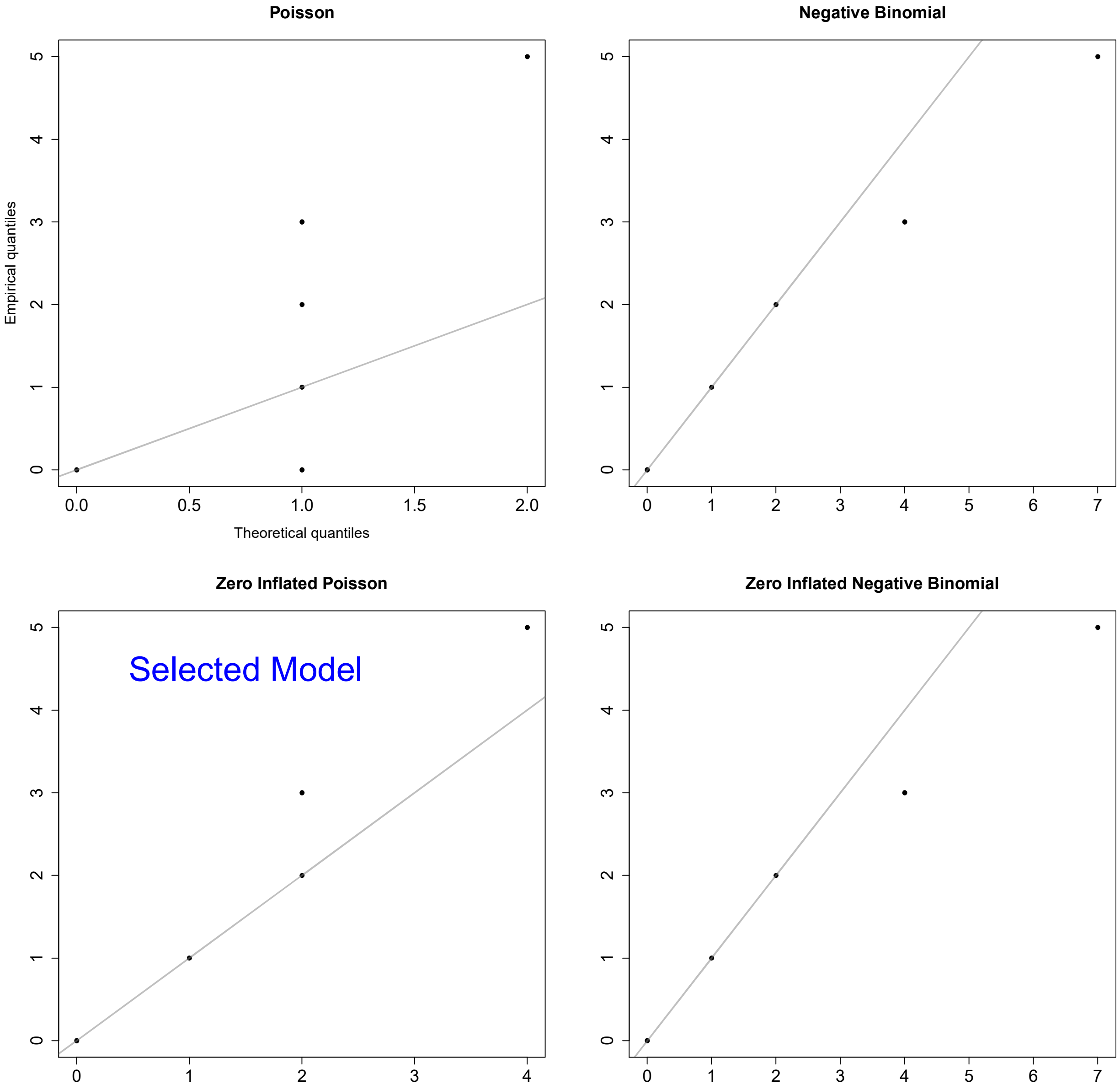

Figure S12. Hygiene Practice: Wash hands (without soap)

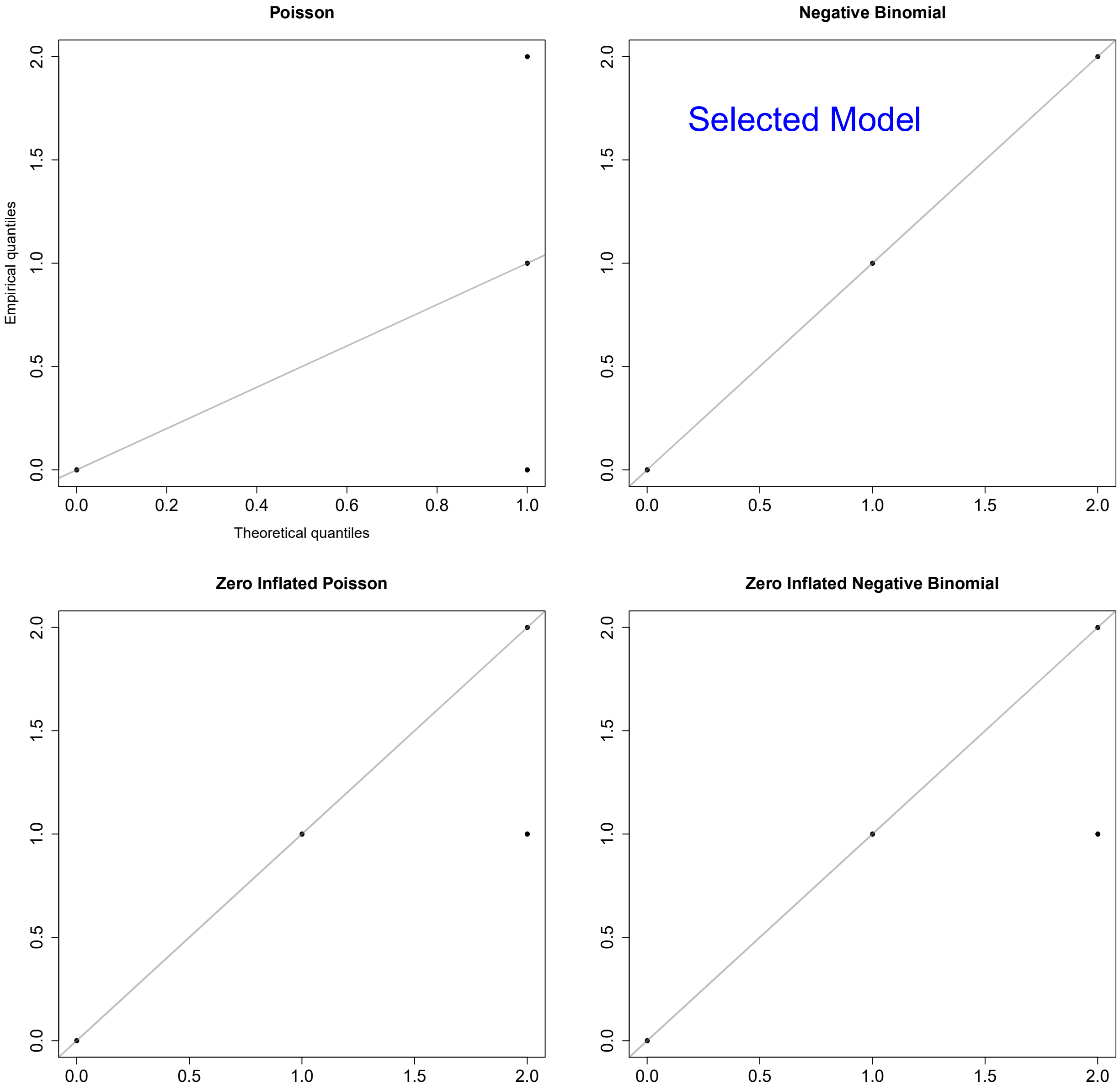

Figure S13. Object-to-Mouth: Hand

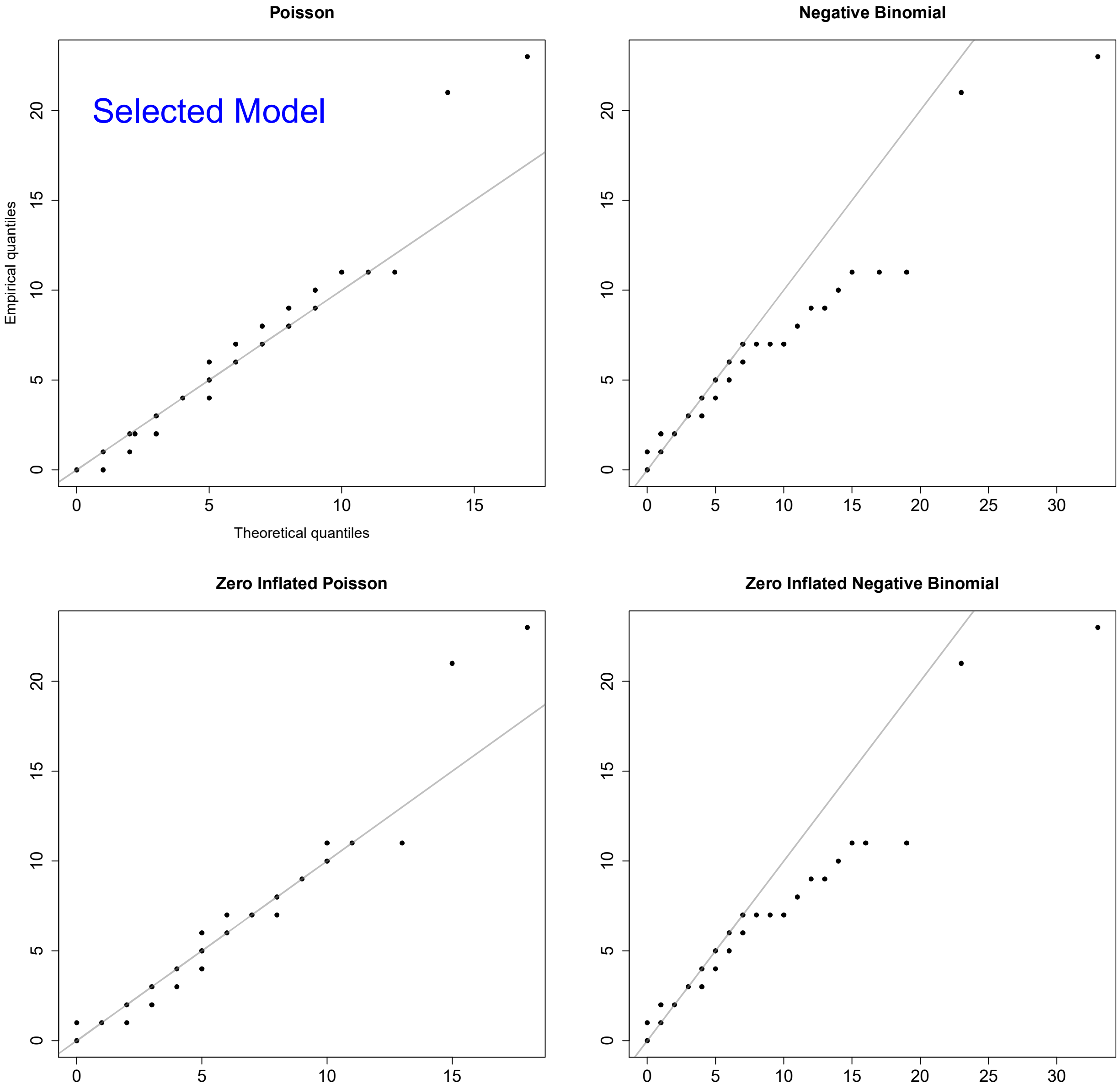

Figure S14. Object-to-Mouth: Unspecified object on the ground

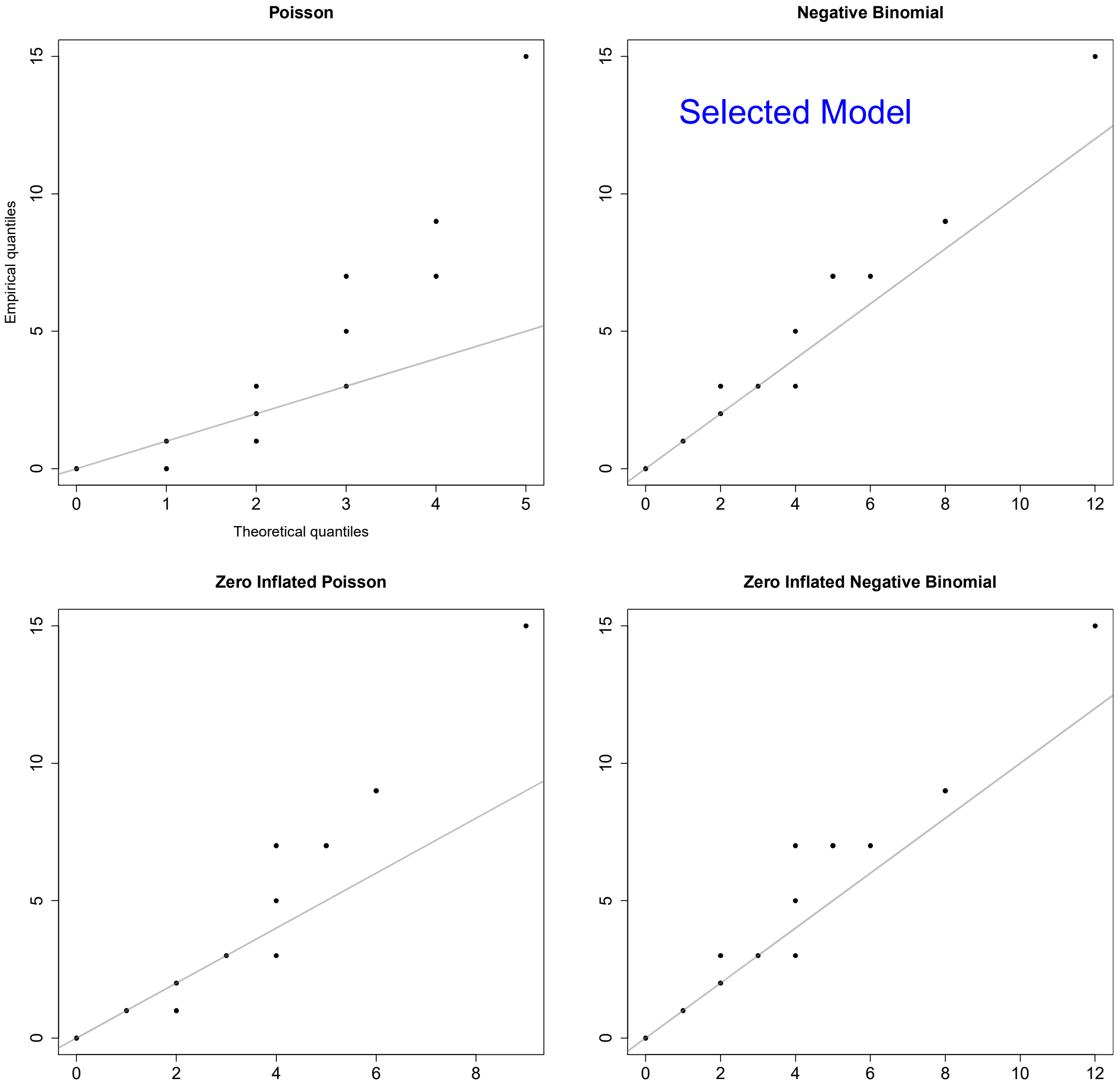

Figure S15. Object-to-Mouth: Unspecified object not on the ground

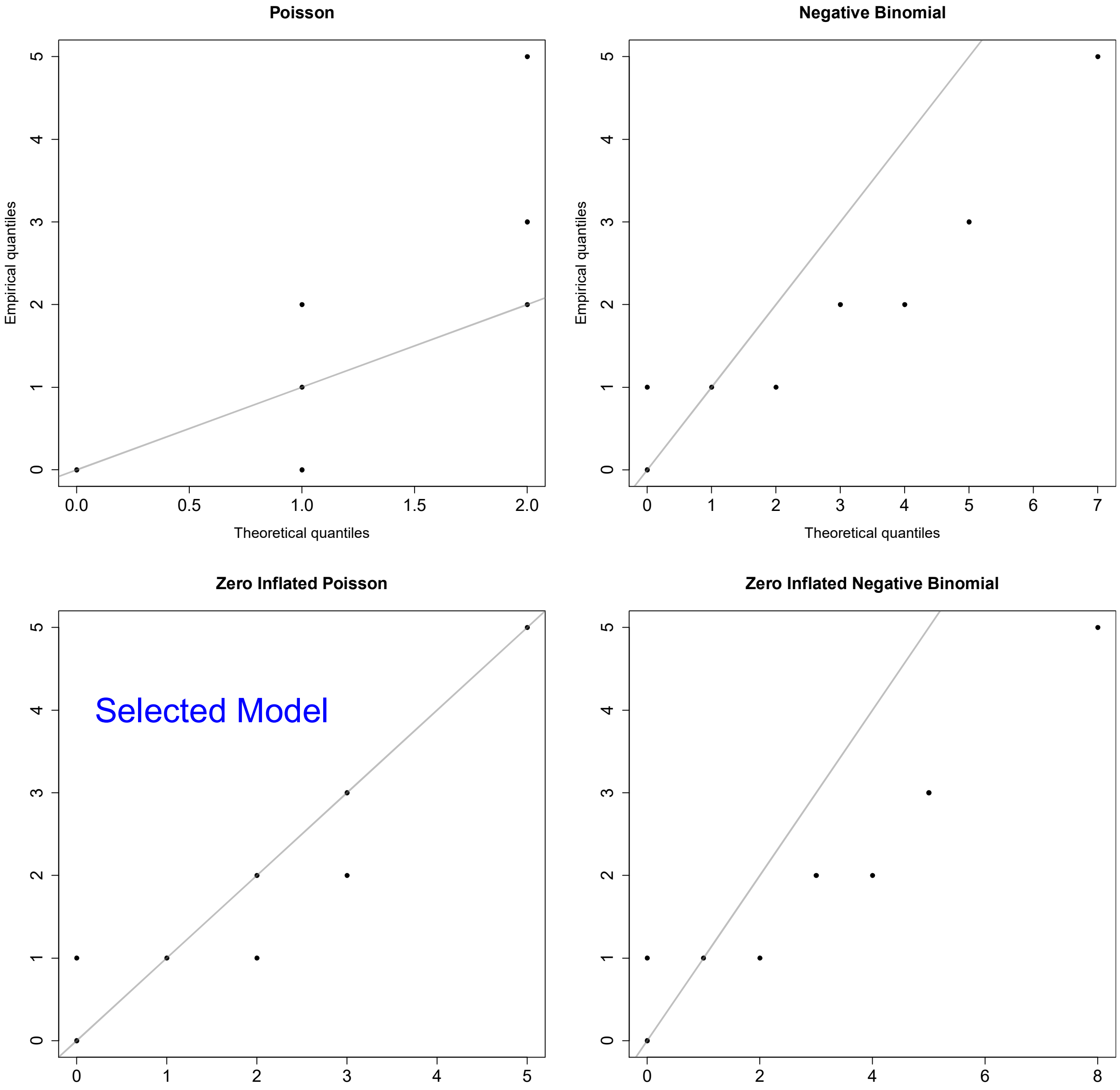

Figure S16. Object-to-Mouth: Soil (geophagia)

*Poisson zero inflated was chosen based on the assumption that not all children engage in geophagia

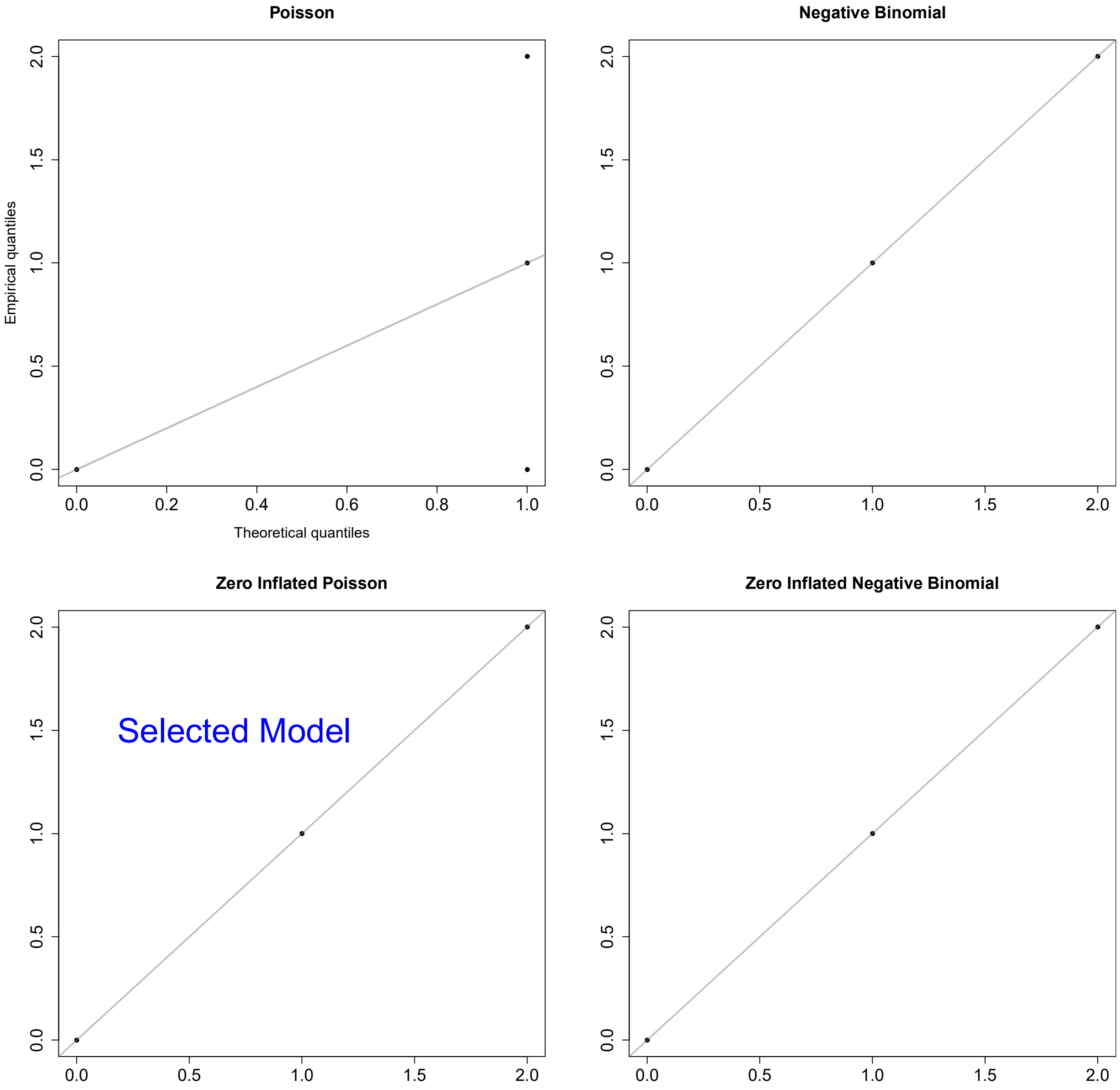

Figure S17. Object-to-Mouth: Surface water (drink)

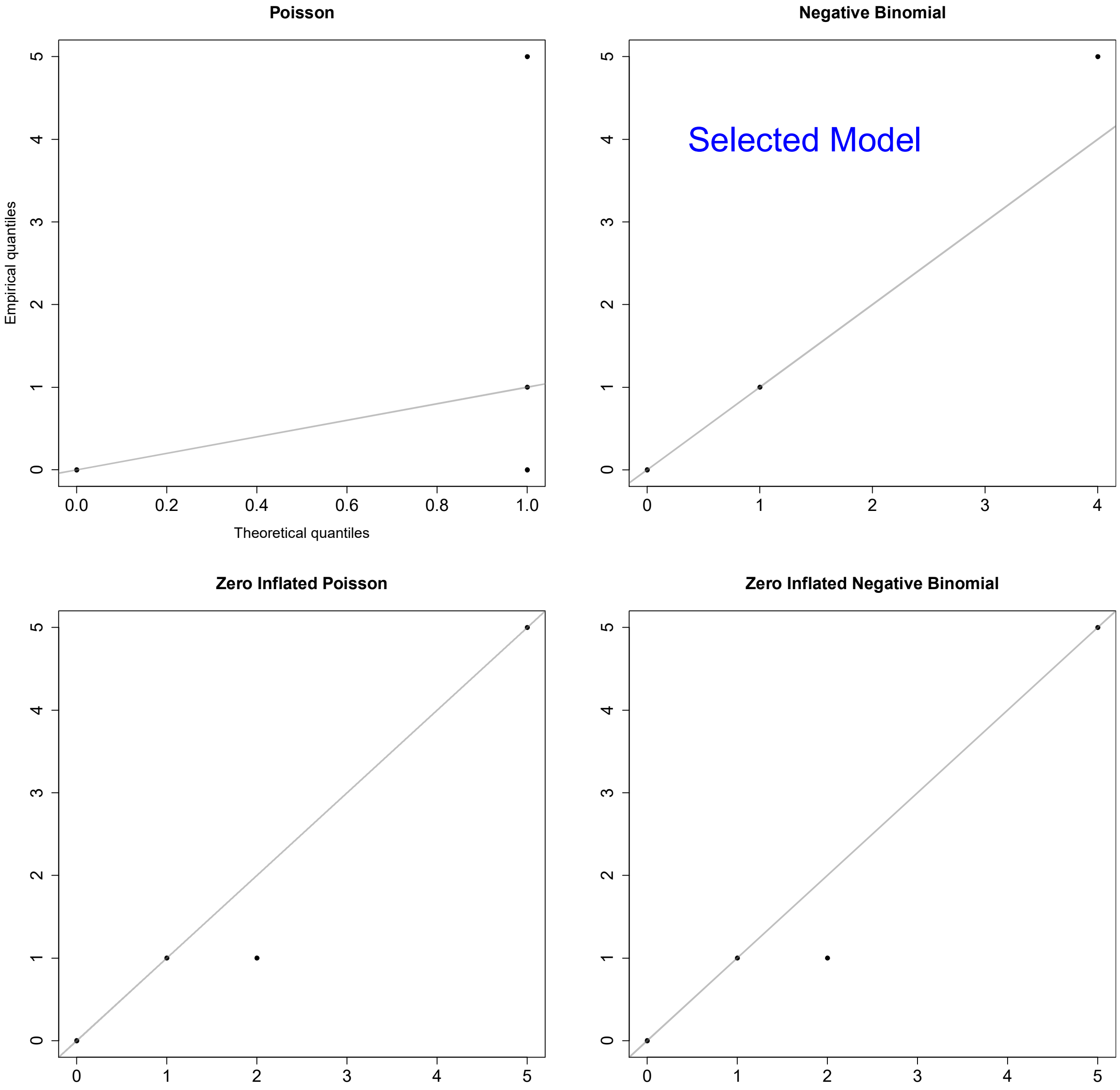

Figure S18. Object-to-Mouth: Trash (metal/glass)

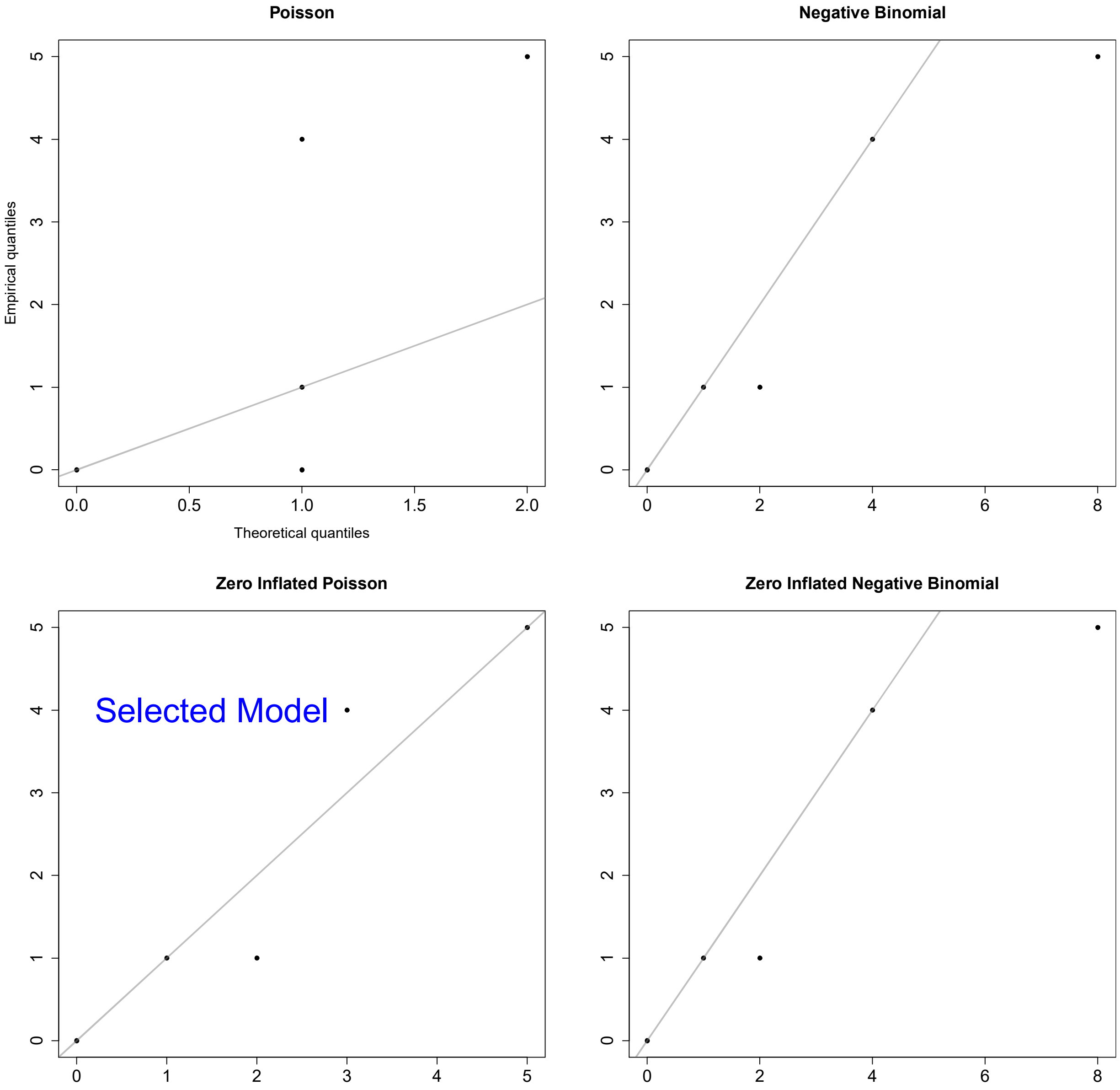

Figure S19. Object-to-Mouth: Trash (plastic/other)

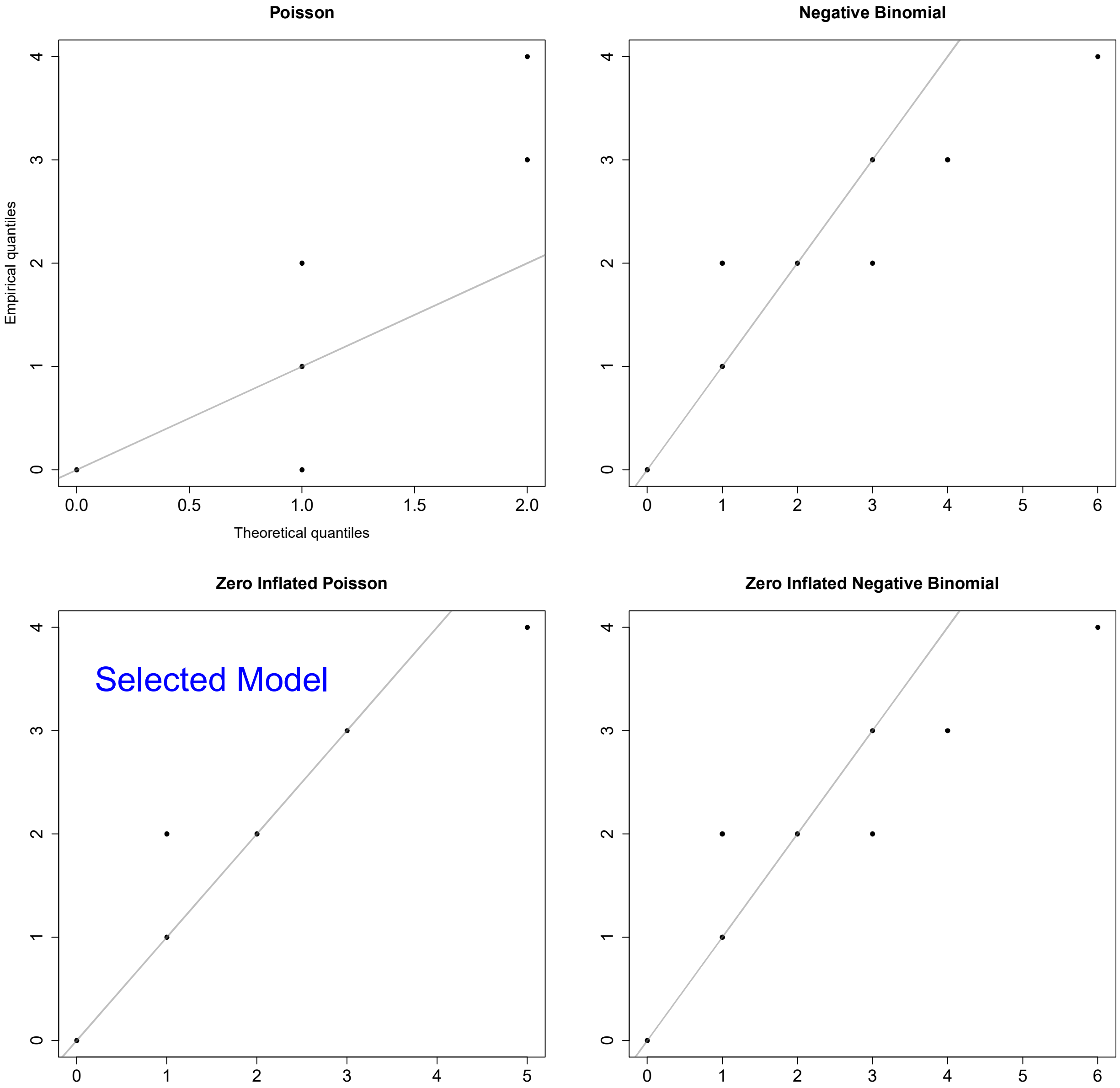

Figure S20. Object-to-Mouth: Eating food on the ground

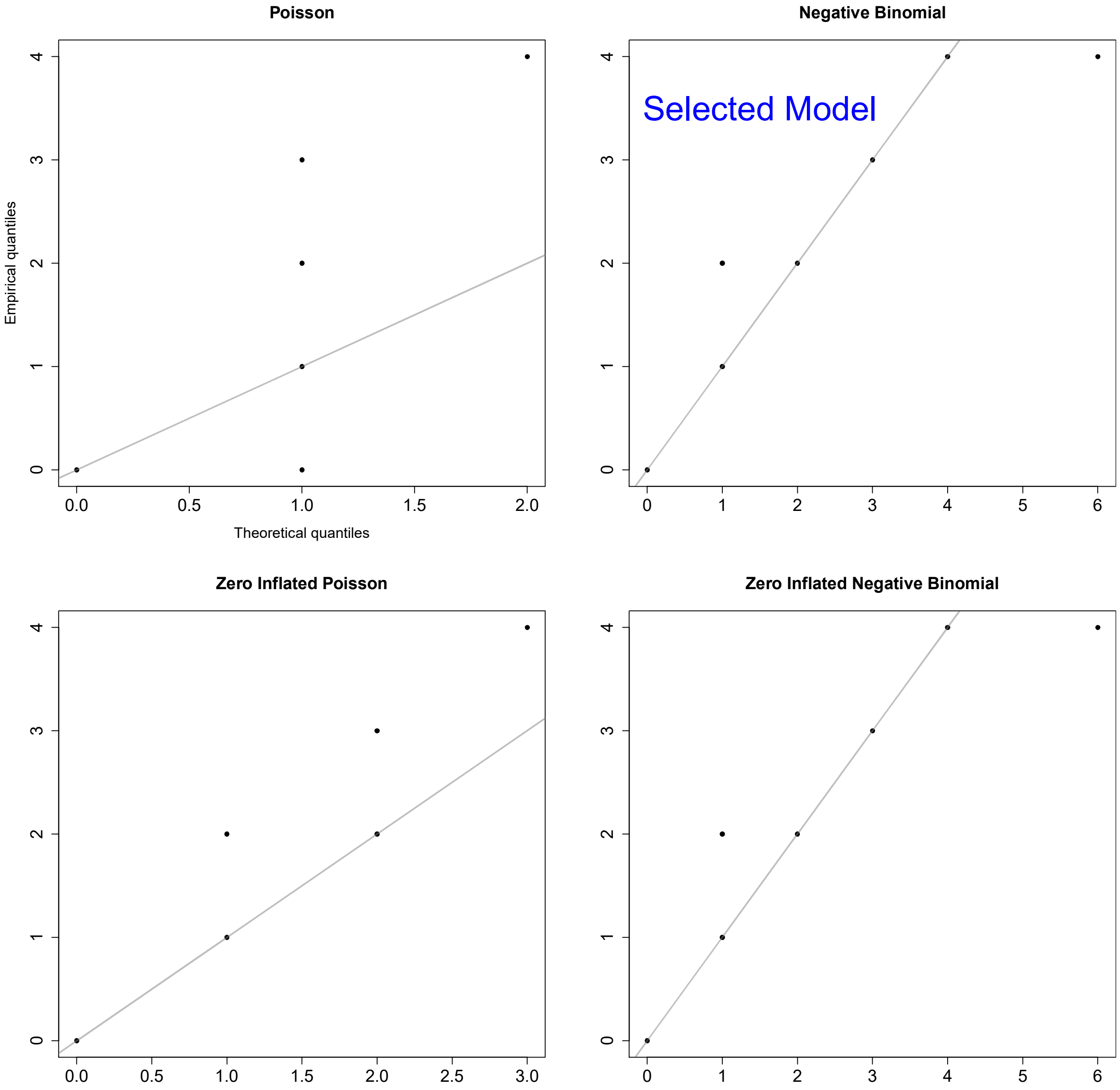

Figure S21. Sanitation Practices: Use of a public latrine

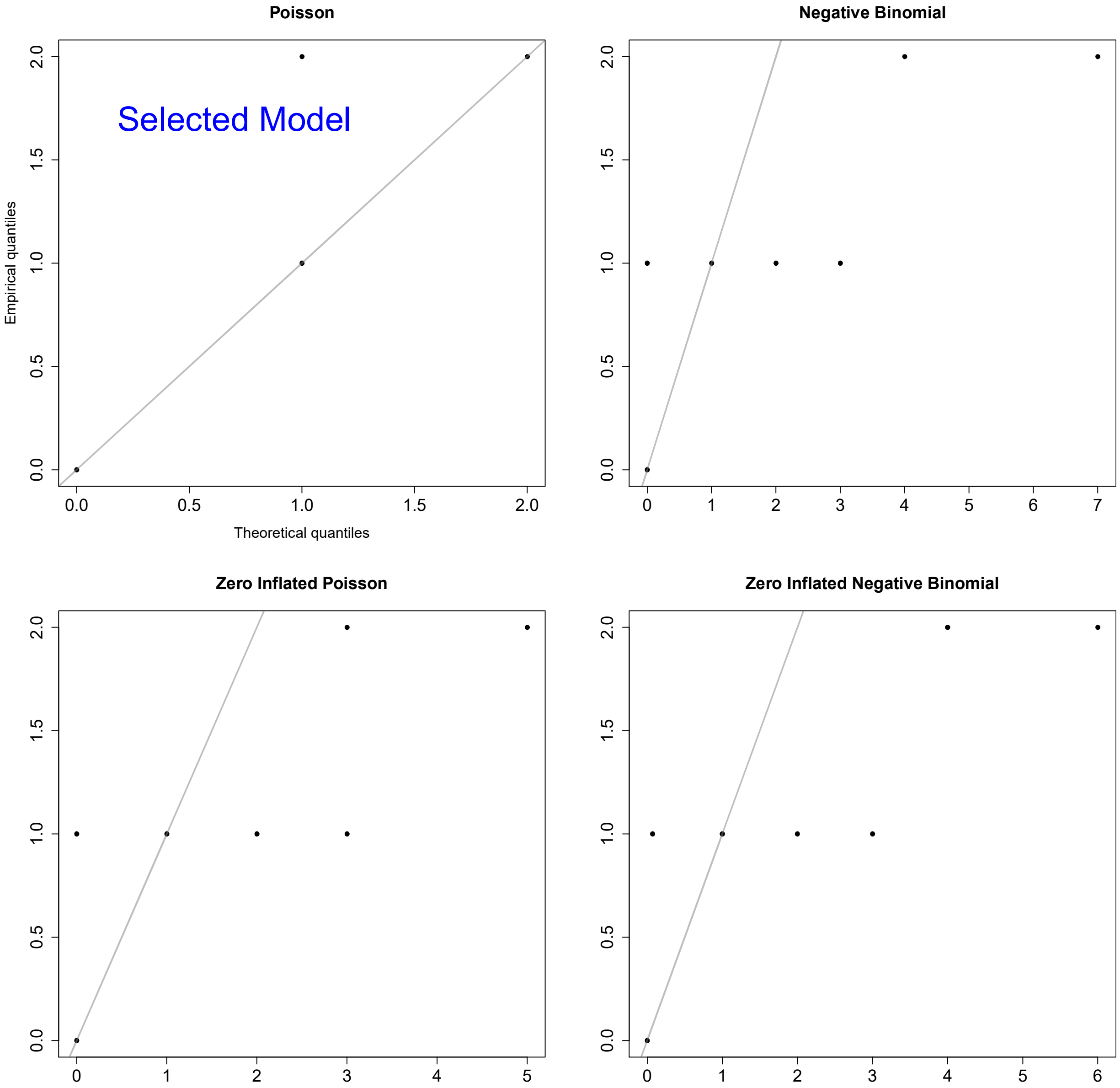

Figure S22. Sanitation Practices: Open defecate

*The Poisson distribution adequately modeled the limited data recorded for open defecation. While the ZIP model might seem more theoretically sound since not all children likely practice open defecation, the limited data did not provide enough information to estimate the probability of engaging in the behavior. Thus, we opted for the simpler model.

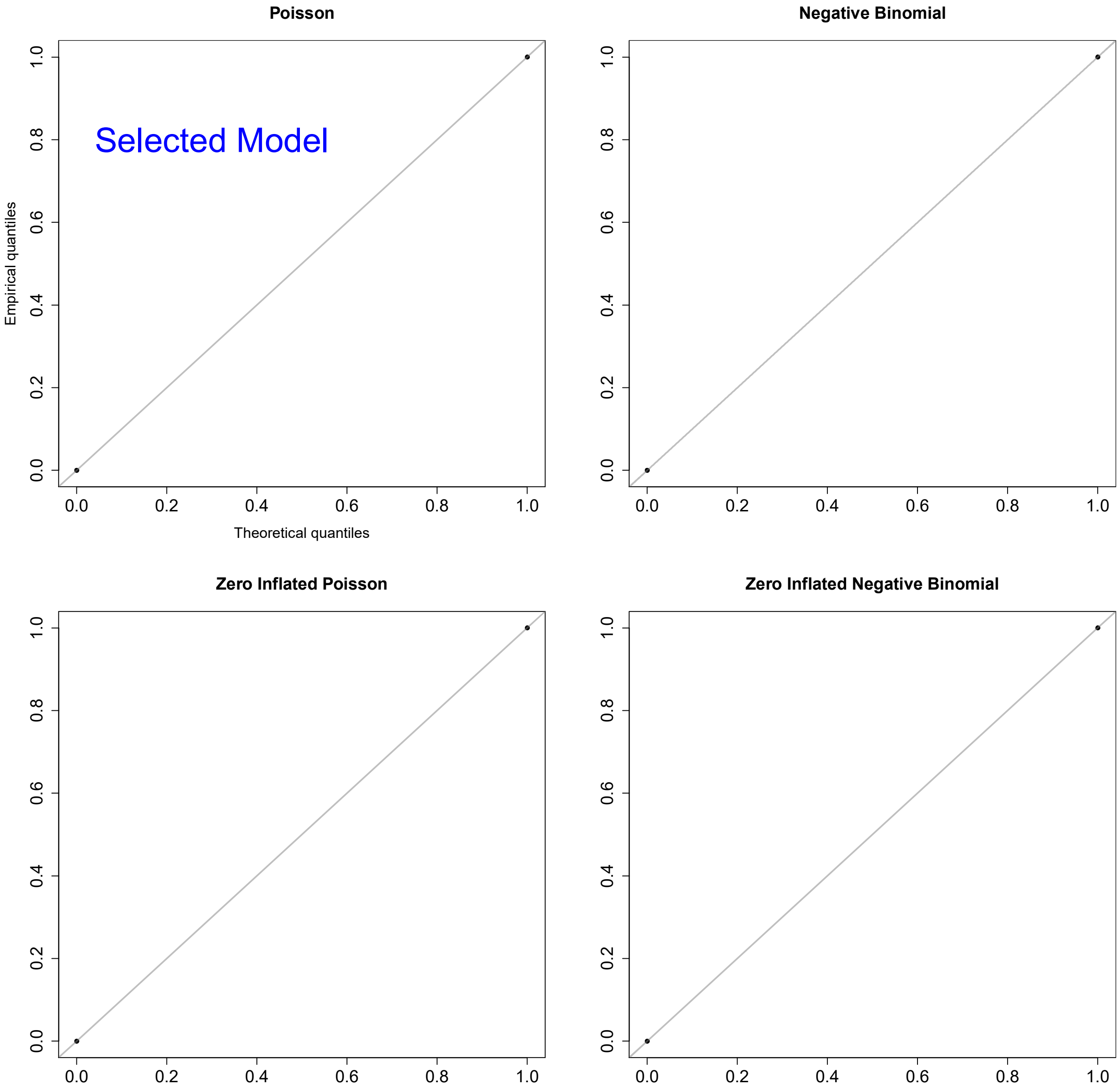

Supplement: Supplementary file 1 [file ijerph-15-01646-s001.pdf]
